# Histone Lactylation Drives WNK1 Expression to Promote Esophageal Squamous Cell Carcinoma Progression Through Histone H3 Ser10 Phosphorylation

**DOI:** 10.1002/advs.78438

**Published:** 2026-10-25

**Authors:** Mingyang Yan, Shaobo Fang, Yanming Song, Lei Zhang, Kexin Zhao, Zhenwei Wang, Yang Shao, Meng Liu, Xinyang Jia, Qinxin Guo, Mee‐Hyun Lee, Guoguo Jin, Zigang Dong

**Affiliations:** ^1^ Department of Pathophysiology School of Basic Medical Sciences Zhengzhou University Zhengzhou Henan China; ^2^ Henan Key Laboratory of Chronic Disease Management Fuwai Central China Cardiovascular Hospital Zhengzhou China; ^3^ China‐US (Henan) Hormel Cancer Institute Zhengzhou Henan China; ^4^ Central China Subcenter of National Center for Cardiovascular Diseases Henan Cardiovascular Disease Center Fuwai Central‐China Cardiovascular Hospital Central China Fuwai Hospital of Zhengzhou University Zhengzhou China; ^5^ Department of Radiology Zhengzhou University People's Hospital & Henan Provincial People's Hospital Zhengzhou China; ^6^ Tianjian Laboratory of Advanced Biomedical Sciences Institute of Advanced Biomedical Sciences Zhengzhou University Zhengzhou Henan China; ^7^ State Key Laboratory of Metabolic Dysregulation & Prevention and Treatment of Esophageal Cancer School of Basic Medical Sciences Zhengzhou University Zhengzhou Henan China

**Keywords:** esophageal squamous cell carcinoma, H3K23la, lactylation, WNK1

## Abstract

Esophageal squamous cell carcinoma (ESCC) lacks effective targeted therapies, and how metabolic reprogramming drives oncogenic signaling remains poorly defined. Here, we identify histone H3 lysine 23 lactylation (H3K23la) as a key epigenetic mediator linking lactate accumulation to tumor progression. H3K23la is upregulated in ESCC and correlates with poor prognosis. Mechanistically, H3K23la promotes WNK lysine deficient protein kinase 1 (WNK1) transcription, which increases histone H3 Ser10 phosphorylation (p‐H3S10) and enhances AKT transcription, thereby driving PI3K‐AKT signaling. This defines a lactate‐H3K23la‐WNK1‐p‐H3S10‐AKT axis connecting metabolism to oncogenic signaling. Importantly, pharmacological inhibition of WNK1 by CD03017 disrupts this pathway and suppresses tumor growth in vitro and in patient‐derived xenograft (PDX) models. In conclusion, our findings define a metabolic‐epigenetic‐signaling axis in ESCC and establish WNK1 as a potential therapeutic target downstream of histone lactylation.

## Graphical abstract

In brief. Yan et al. demonstrated that the excess lactate produced by the enhanced glycolysis pathway in esophageal cancer can lactylate H3K23, H3K23la can be enriched in the *WNK1* promoter region to promote WNK1 expression, thereby increasing the phosphorylation of the p‐H3 Ser10 site, and p‐H3S10 can be enriched in the *AKT1* promoter region to promote AKT expression, thereby enhancing the activation of the AKT pathway and promoting the occurrence of esophageal cancer. CD03017 can inhibit the WNK1 kinase activity and thus block the lactate–H3K23la–WNK1–p‐H3S10–AKT axis to inhibit the occurrence of ESCC.

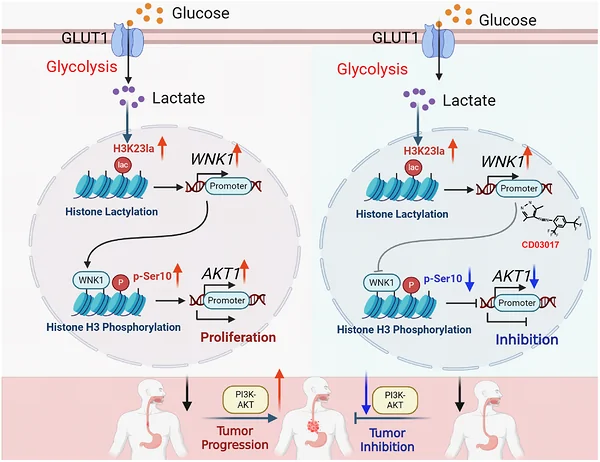

## 1 Introduction

Esophageal cancer is one of the most common malignancies worldwide and is associated with a poor prognosis, with a 5‐year survival rate of less than 20% [1, 2, 3, 4, 5]. Esophageal squamous cell carcinoma (ESCC), the predominant subtype, lacks effective targeted therapies, and current treatment strategies‐including surgery, radiotherapy, and chemotherapy‐provide limited clinical benefit [6, 7, 8]. Despite decades of research, survival outcomes have improved only marginally, underscoring an urgent need to identify actionable molecular mechanisms and novel therapeutic targets in ESCC.

Metabolic reprogramming is a hallmark of cancer, with the Warburg effect representing a shift toward aerobic glycolysis even under normoxic conditions [9, 10, 11, 12, 13]. This metabolic adaptation results in excessive lactate accumulation, which has long been considered a metabolic byproduct but is now recognized as an active signaling molecule capable of regulating gene expression [14, 15, 16]. Notably, lactate has been shown to directly influence chromatin states through histone lactylation, a recently identified epigenetic modification that links cellular metabolism to transcriptional control [16, 17, 18, 19]. However, a central unresolved question remains: how lactate‐driven epigenetic modifications are functionally translated into oncogenic signaling programs in ESCC.

Recent studies have begun to explore histone lactylation at multiple lysine residues [20, 21, 22], yet the functional specificity of different sites in various cancers remains poorly defined [19, 21, 23, 24, 25, 26]. Among these, histone H3 lysine 23 lactylation (H3K23la) has received little attention, and its role in ESCC is largely unknown. Whether H3K23la serves as a critical effector linking glycolytic metabolism to oncogenic transcriptional programs, and how it integrates into signaling networks that drive tumor progression, remains to be elucidated. Protein kinases are attractive therapeutic targets in cancer due to their central roles in regulating tumor‐associated signaling networks, and several kinase inhibitors have shown clinical efficacy [27, 28]. WNK lysine deficient protein kinase 1 (WNK1) is a chromatin‐regulated kinase that is closely related to tumor progression and whose function has not been fully elucidated [29, 30, 31, 32]. Whether WNK1 serves as a key mediator linking metabolic alterations to oncogenic signaling in ESCC warrants further investigation.

In this study, we investigated the functional role of H3K23la in ESCC and its contribution to tumor progression. We found that H3K23la is elevated in ESCC and promotes tumor growth by transcriptionally activating WNK1. Mechanistically, WNK1‐mediated H3S10 phosphorylation links histone lactylation to AKT signaling activation, establishing a metabolic‐epigenetic‐signaling axis in ESCC. Furthermore, pharmacological inhibition of WNK1 by CD03017 suppresses tumor growth in vitro and in PDX models, highlighting WNK1 as a potential therapeutic target in ESCC.

## 2 Results

### 2.1 Upregulated H3K23la Predicts Poor Prognosis in Esophageal Cancer Patients

Cancer cells are characterized by a significant alteration in cellular metabolism, most notably the shift toward glycolysis even under aerobic conditions, a phenomenon known as the Warburg effect [10, 33, 34]. This metabolic reprogramming supports rapid tumor growth and survival [9]. To better characterize this metabolic shift, we performed Gene Set Enrichment Analysis (GSEA) using Kyoto Encyclopedia of Genes and Genomes (KEGG) gene sets on publicly available transcriptomic datasets, comparing tumor tissues with matched adjacent normal tissues across multiple cancer types. This analysis showed that the “Glycolysis/Gluconeogenesis” pathway was significantly enriched in ESCC, STAD, and LIHC tumors relative to their corresponding normal controls (Figure 1A), underscoring the prominent activation of glycolysis during metabolic reprogramming in these malignancies. Further analysis at the gene expression level revealed that several key glycolytic enzymes, such as ALDO, GAPDH, ENO1, PFK‐1, and LDHA, are generally upregulated in tumor tissues, supporting the enhanced glycolytic metabolism observed in cancer cells (Figure 1B). Using a lactate assay kit, we found that lactate levels were significantly elevated in the plasma of ESCC patients compared with normal patients (*p* = 0.0012), further confirming enhanced glycolytic activity at the metabolite level (Figure 1C,D). Furthermore, lactate transporters, notably monocarboxylate transporter 1 (MCT1), were significantly upregulated in ESCC tissues (Figure 1E). This upregulation likely facilitates lactate efflux, mitigating intracellular acid stress to sustain active glycolysis. To further explore this metabolic shift, we found that the lactate concentration in the culture supernatant of ESCC cells was significantly increased (Figure 1F), providing additional evidence for enhanced glycolysis and lactate accumulation in ESCC.

**FIGURE 1:**
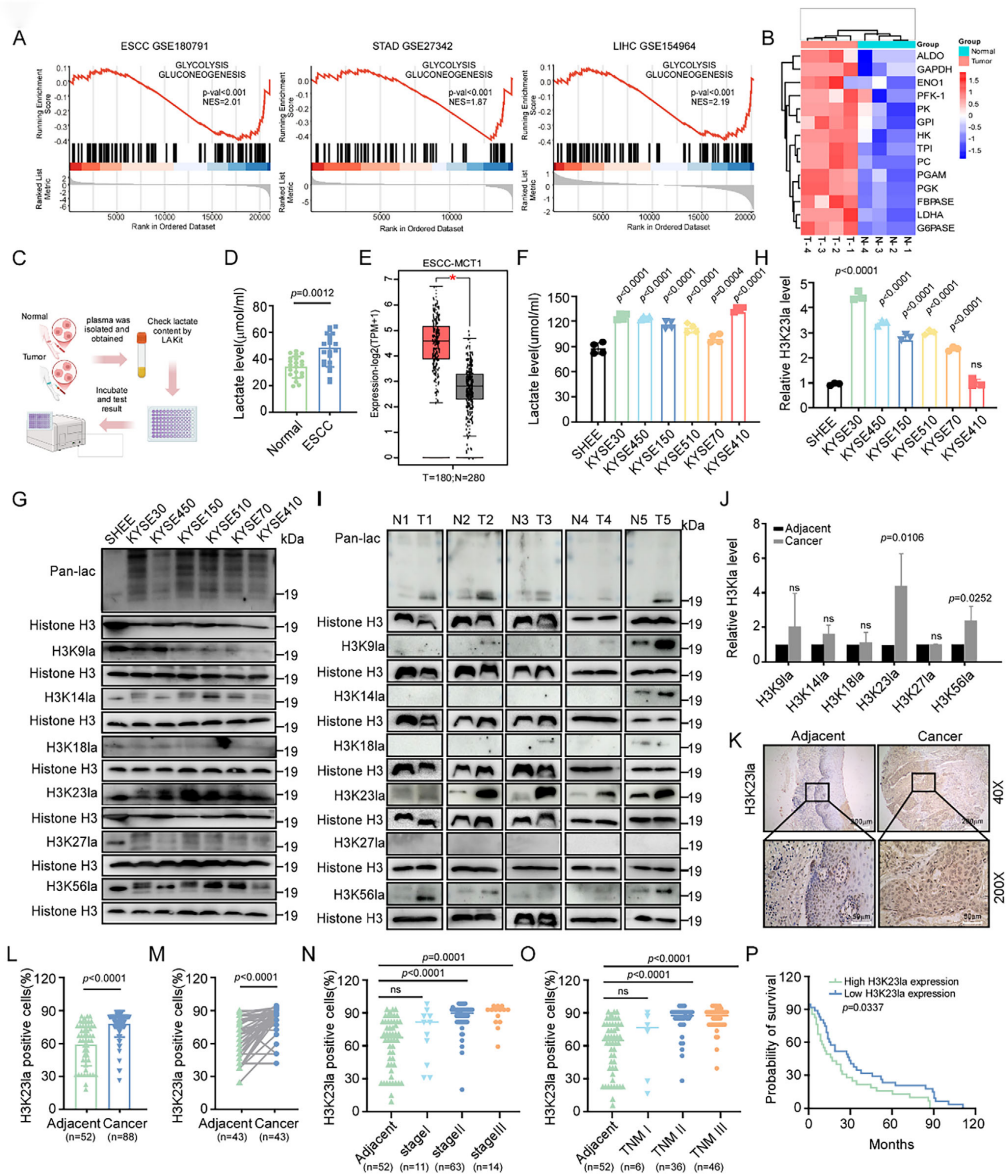
Elevated lactate levels and histone lactylation are associated with poor prognosis in patients with ESCC. (A) The enrichment of the glycolysis‐gluconeogenesis pathway in KEGG was analyzed in ESCC, STAD, and LIHC through GSEA. (B) Heatmap analysis was performed to examine the enrichment of glycolysis‐related metabolic enzymes in tumor and adjacent non‐tumor tissues. (C) The schematic diagram illustrates the measurement of lactate levels in the serum of ESCC patients and healthy individuals. (D) Lactate levels in the serum of ESCC patients and healthy individuals were measured using a lactate assay kit. (E) The expression of MCT1 in tumor and adjacent non‐tumor tissues of ESCC was analyzed using the TCGA database. (F) Lactate levels in the supernatants of ESCC cell lines and SHEE cells were measured using a lactate assay kit (n = 4). (G,H) Histone H3 lactylation at different sites in ESCC cell lines and SHEE cells were detected using the WB method. The results were quantitatively analyzed (n = 3). (I,J) Histone H3 lactylation at different sites in tumor and adjacent non‐tumor tissues of ESCC was detected using the WB method, and the results were quantitatively analyzed (n = 3). (K) Representative IHC staining images showing H3K23la levels in human esophageal cancer tissues. (L) IHC staining score analysis of H3K23la expression in unpaired tumor and adjacent normal esophageal tissues. (M) Comparison of IHC staining scores for H3K23la levels between tumor and paired normal tissues. (N) IHC staining scores of H3K23la levels in tumors at different clinical stages. (O) H3K23la IHC scores according to pathological TNM stage. (P) Kaplan–Meier analysis of overall survival according to H3K23la expression. Data are presented as mean ± SD from three independent biological experiments unless otherwise indicated. Statistical significance was determined using two‐tailed unpaired Student's t‐test (D, J, and L), paired two‐tailed Student's t‐test (M), one‐way ANOVA followed by Tukey's multiple‐comparison test (F, H, N, and O), and the log‐rank (Mantel‐Cox) test (P). MCT1 expression in TCGA datasets (E) was analyzed using the statistical method implemented in the corresponding database. GSEA significance (A) was evaluated using permutation testing with normalized enrichment score (NES) and nominal P value. Exact P values are provided in the Source Data where applicable. ns, no significant difference.

Building on these observations, we next asked whether the glycolytic reliance and lactate buildup in ESCC confer a growth advantage to tumor cells. Pharmacological inhibition of glycolysis with Dichloroacetate (DCA), Oxamate, or 2‐deoxy‐D‐glucose (2‐DG) markedly suppressed the proliferation of KYSE30 and KYSE450 cells in a dose‐ and time‐dependent manner, underscoring the dependency of ESCC cells on glycolytic metabolism for sustained growth (Figure S1A–C). MTT results indicated that DCA, Oxamate and 2‐DG were non‐toxic to SHEE cells (Figure S1D). Consistent results from colony formation assays showed that increasing concentrations of 2‐DG, DCA, and Oxamate significantly reduced colony numbers in both cell lines (Figure S1E–J). MTT results indicated that co‐treatment with 2‐DG (1 mM) and sodium lactate (NaLa) (10 mM) partially reversed the inhibitory effect of 2‐DG, suggesting that lactate may play a role in promoting the proliferation of ESCC cells (Figure S1K). Moreover, the addition of NaLa (10 mM) markedly restored the colony‐forming ability of cells treated with 2‐DG (1 mM) (Figure S1L,M). Collectively, these findings demonstrate that ESCC cell proliferation is associated with glycolytic metabolism and further imply that lactate accumulation serves as a key growth‐promoting output of glycolysis.

Lactate, traditionally regarded as a metabolic waste product of glycolysis, is now recognized as a key signaling molecule capable of regulating epigenetic modifications through histone lactylation [16]. Recent studies have shown that intracellular accumulation of lactate can drive the lactylation of lysine residues on histones, thereby influencing gene expression and tumor progression [17]. To investigate whether lactate accumulation affects epigenetic modifications in ESCC, we evaluated both global and site‐specific histone lactylation levels in ESCC cell lines and tumor tissues using Western blotting (WB). Compared to normal controls, ESCC cells and tumor tissues exhibited markedly increased levels of total lactylation (Pan‐lac) and multiple site‐specific histone lactylation marks, including H3K9la, H3K14la, H3K18la, and particularly H3K23la (Figure 1G,I). Quantitative analysis revealed that H3K23la was consistently elevated across multiple ESCC cell lines and tumor tissues (Figure 1H,J), suggesting it is a key lactylation target in ESCC. To investigate the functional significance of individual histone H3 lactylation sites in ESCC, we generated H3K9A, H3K14A, H3K18A, and H3K23A mutants. Functional assays demonstrated that depletion of histone H3 markedly suppressed ESCC cell proliferation and colony‐forming capacity. Rescue experiments further revealed that re‐expression of the H3K9A, H3K14A, or H3K18A mutants effectively restored the impaired proliferative and clonogenic phenotypes induced by histone H3 knockdown. In contrast, re‐expression of the H3K23A mutant failed to rescue these defects (Figure S1N–P). These findings indicate that lactylation at H3K23 is indispensable for sustaining the proliferative capacity of ESCC cells. All the results indicate that lactate may partially compensate for impaired glycolysis and contribute to the survival and proliferation of ESCC cells.

To validate this observation, we performed immunohistochemistry (IHC) analysis on a commercial tissue microarray of ESCC samples, confirming that H3K23la expression was markedly higher in tumor tissues compared to adjacent normal tissues (Figure 1K–M). Patients with high H3K23la expression exhibited higher histological grade, advanced TNM stage, and poorer prognosis (Figure 1N–P). Meanwhile, we also performed multivariate Cox regression analysis, which showed that H3K23la is an independent prognostic factor for ESCC (Table S1). These findings suggest that lactate‐mediated histone lactylation plays a significant role in ESCC progression and may serve as a novel biomarker for the disease. In line with the in vitro findings, we next assessed the impact of glycolytic blockade in an ESCC xenograft model. Treatment of LEG643 tumor‐bearing mice with 2‐DG (500 mg/kg; i.p.) markedly reduced tumor size, weight, and growth rate compared with vehicle controls, whereas co‐administration of NaLa (150 mg/kg; i.p.) partially restored these parameters (Figure S1Q,R). IHC analysis of the PDX model revealed that revealed that 2‐DG‐treated tumors displayed diminished cellularity on IHC staining, a sharp decline in the proliferative marker Ki67, and a pronounced reduction in the histone‐lactylation mark H3K23la; All the effects were partially reversed by NaLa treatment (Figure S1S,T). We also performed in vivo rescue experiments using cell‐derived xenograft (CDX) models. ESCC cells with histone H3 knockdown were reconstituted with either wild‐type H3 or the lactylation‐deficient H3K23A mutant. Re‐expression of wild‐type H3, but not H3K23A, restored tumor growth in vivo, supporting a role for the H3K23 residue and its potential lactylation in ESCC tumorigenesis (Figure S1U,V).

Together, these findings demonstrate that enhanced glycolysis and lactate accumulation drive ESCC progression not only by promoting tumor cell proliferation but also by inducing epigenetic reprogramming through histone lactylation, particularly at H3K23la, which may serve as both a functional effector and a prognostic biomarker in ESCC.

### 2.2 H3K23la Enrichment at the *WNK1* Promoter Promotes WNK1 Transcription in ESCC

Histone H3 is a core component of nucleosomes and plays an important role in chromatin organization and transcriptional regulation through post‐translational modifications [35, 36, 37, 38, 39]. To explore the transcriptional regulatory role of H3K23la in ESCC, we first performed CUT‐Tag sequencing in KYSE450 cells to map genomic regions enriched for H3K23la (Figure 2A). Raw sequencing reads were subjected to quality assessment using FastQC and processed with fastp to remove adapter sequences and low‐quality reads. The filtered reads were aligned to the human reference genome using Bowtie2, and H3K23la‐enriched regions were identified by peak calling with MACS3, followed by genomic annotation and analysis of peak distribution relative to transcription start sites (TSSs).The sequencing results revealed that H3K23la peaks were primarily enriched at promoter regions, with enriched binding occurring mainly outside 1000 base pairs from the transcription start site (TSS), indicating that H3K23la may function as a transcriptional activator during the transcription process (Figure 2B–D). Further analysis revealed that H3K23la most notably binds to the promoter region of the *WNK1* gene located on chromosome 12, suggesting a potential regulatory association between H3K23la and WNK1 transcription in ESCC cells (Figure 2E). To further explore the possible transcriptional regulatory role of H3K23la, we integrated the binding analysis with transcriptomic data. Specifically, we focused on differentially expressed genes (DEGs) identified in our RNA‐seq dataset using a threshold of |log2 fold change| > 1, which represents genes showing substantial transcriptional changes under the experimental condition.

**FIGURE 2:**
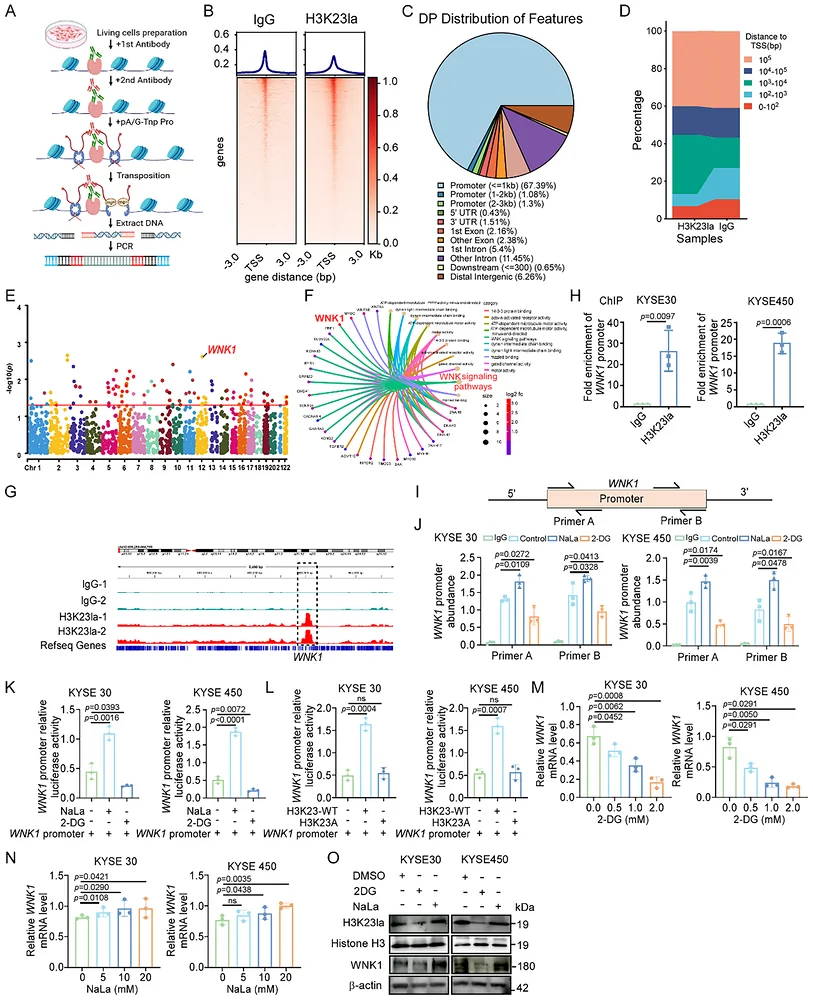
H3K23la activates WNK1 transcription in ESCC. (A) Schematic diagram of CUT‐Tag process. (B) The distribution of H3K23la on the genome. (C) KEGG analysis of the promoter region of the H3K23la distribution. (D) The distance to the closest gene TSS of all regions and the new GAIN and LOSS regions for H3K23la enrichment. (E) The distribution of regions for H3K23la enrichment across chromosomes. (F) Top 10 KEGG pathway‐associated DEGs related to the H3K23la enrichment regions. DEGs are shown in a color gradient based on their log2fold change (FC) values. (G) WashU Epigenome Browser tracks showing H3K23la (red) signals of representative upregulated WNK1 genes in KYSE450 cell line, each with two replicates. The H3K23la GAIN regions are shaded in red. (H) ChIP‐qPCR analysis of H3K23la enrichment at the *WNK1* promoter (n = 3). (I,J) Schematic of the *WNK1* promoter region indicating the positions of Primer A and Primer B used in subsequent ChIP‐qPCR assays. (K) Luciferase reporter assay showing *WNK1* promoter activity in ESCC cells (n = 3). (L) Luciferase reporter assay showing that mutation of H3K23 abrogates NaLa‐induced *WNK1* promoter activation in ESCC cells (n = 3). (M,N) qRT‐PCR analysis of *WNK1* mRNA levels in ESCC cells treated with increasing concentrations of 2‐DG or NaLa (n = 3). (O) WB analysis of WNK1 and H3K23la protein levels in ESCC cells under treatment with DMSO, NaLa, or 2‐DG. Data are presented as mean ± SD from three independent biological experiments unless otherwise indicated. Statistical significance was determined using two‐tailed unpaired Student's t‐test (H) and one‐way ANOVA followed by Tukey's multiple‐comparison test (I–M). CUT‐Tag peak calling, differential enrichment, and KEGG pathway enrichment analyses (B–G) were performed using standard bioinformatic pipelines with false discovery rate (FDR) correction where applicable. Exact P values are provided in the Source Data whenever possible. ns, no significant difference.

Based on this criterion, we obtained a set of DEGs that were subsequently subjected to KEGG pathway enrichment analysis to identify biological pathways potentially associated with H3K23la‐related transcriptional regulation. The enrichment analysis revealed that 25 key DEGs were significantly enriched within the top 10 KEGG pathways, indicating that these genes may participate in several biologically relevant signaling processes. Among these enriched pathways, the WNK signaling pathway was prominently represented. Notably, WNK1, which had been identified in the H3K23la binding analysis as a gene whose promoter region shows strong H3K23la enrichment, was also included among the genes significantly enriched in this pathway (Figure 2F). The convergence of these two independent analyses‐chromatin binding profiling and transcriptomic pathway enrichment‐suggests a potential link between H3K23la‐associated chromatin binding and the regulation of WNK1‐related signaling pathways.

To identify the downstream targets of H3K23la, we further analyzed the results of CUT‐Tag. The results revealed a prominent enrichment of H3K23la at the promoter region of the *WNK1* gene on chromosome 12 (Figure 2G), suggesting that WNK1 may serve as a direct transcriptional target of H3K23la. ChIP assays further confirmed the enrichment of H3K23la at the *WNK1* promoter in ESCC cells (Figure 2H). Meanwhile, ChIP‐qPCR analysis showed that NaLa treatment significantly increased H3K23la enrichment at the *WNK1* promoter, whereas 2‐DG treatment significantly decreased H3K23la enrichment (Figure 2I,J). To determine whether cellular lactate levels regulate H3K23la‐mediated WNK1 transcriptional activation, luciferase reporter assays were performed. NaLa (10 mM) treatment increased *WNK1* promoter activity, whereas 2‐DG (1 mM) treatment reduced this activity (Figure 2K). Similar results were consistently observed across multiple ESCC cell lines and different *WNK1* promoter constructs (Figure S2A–E), indicating that cellular lactate levels dynamically regulate H3K23la‐dependent WNK1 transcriptional activation. Because lysine 23 of histone H3 can also undergo acetylation, we next examined the association between H3K23 acetylation (H3K23ac) and the *WNK1* promoter. In contrast to H3K23la, H3K23ac showed no detectable enrichment at the *WNK1* promoter (Figure S2F,G), suggesting that regulation of WNK1 is specific to H3K23 lactylation rather than acetylation.

To further investigate the contribution of lactate uptake to WNK1 transcriptional activation, cells were treated with NaLa (10 mM) in the presence or absence of the MCT1 inhibitor AZD3965 (100 nM), followed by dual‐luciferase reporter assays. NaLa significantly increased *WNK1* promoter activity, whereas AZD3965 largely abolished this effect (Figure S2H). Consistently, WB analysis demonstrated that AZD3965 effectively blocked the NaLa‐induced upregulation of WNK1 protein expression (Figure S2I). These findings indicate that cellular lactate uptake and the consequent increase in H3K23la are required for activation of WNK1 transcription. To further validate the functional importance of the H3K23la‐binding region, the corresponding binding sequence within the *WNK1* promoter was deleted. Under these conditions, neither NaLa (10 mM) nor 2‐DG (1 mM) altered activation of WNK1 transcription (Figure S2J–L), indicating that this promoter region is essential for lactate‐responsive H3K23la binding. Importantly, mutation of H3K23 to alanine abolished the H3K23‐mediated enhancement of WNK1 promoter activity (Figure 2L). Consistently, luciferase reporter assays showed that the H3K23A mutation abolished the responsiveness of *WNK1* promoter activity to NaLa (10 mM) and 2‐DG (1 mM) treatment (Figure S2M,N). These results demonstrate that H3K23 lactylation is required for lactate‐dependent transcriptional activation of WNK1. At the transcriptional level, 2‐DG decreased *WNK1* mRNA expression in a dose‐dependent manner (Figure 2M), whereas NaLa significantly increased *WNK1* mRNA levels in both ESCC cell lines (Figure 2N). Consistent with the above findings, WB analysis showed that 2‐DG (1 mM) reduced H3K23la and WNK1 protein levels, whereas NaLa (10 mM) increased both in KYSE30 and KYSE450 cells (Figure 2O), further supporting lactate‐dependent regulation of WNK1 expression through H3K23la.

Collectively, these findings demonstrate that elevated intracellular lactate promotes p300/CBP‐mediated H3K23 lactylation, enhances H3K23la occupancy at the *WNK1* promoter, and subsequently activates WNK1 transcription and protein expression. Conversely, inhibition of glycolysis, blockade of lactate uptake, disruption of the H3K23 lactylation site, or deletion of the H3K23la‐responsive promoter region all impaired this regulatory axis. Together, these results identify H3K23 lactylation as a metabolic epigenetic signal that directly activates WNK1 transcription, thereby linking lactate metabolism to WNK signaling and potentially promoting ESCC progression.

### 2.3 High WNK1 Expression Indicates Poor Prognosis and its Knockdown Inhibits ESCC Growth In Vitro and In Vivo

To investigate the role of WNK1 in ESCC, we first conducted IHC staining on a tissue microarray containing both tumor and adjacent normal tissues to assess the expression levels of WNK1. IHC analysis revealed that WNK1 expression was significantly upregulated in ESCC tumor tissues compared to adjacent normal tissues (Figure 3A–C), suggesting that WNK1 may play a role in the progression of ESCC. Further analysis indicated that elevated WNK1 expression was closely associated with clinical factors, including stage (Figure 3D), tumor size (Figure 3E), local metastasis (Figure 3F), and poorer survival outcomes (Figure 3G), highlighting WNK1 as a potential tumor marker closely linked to the tumor characteristics of ESCC.

**FIGURE 3:**
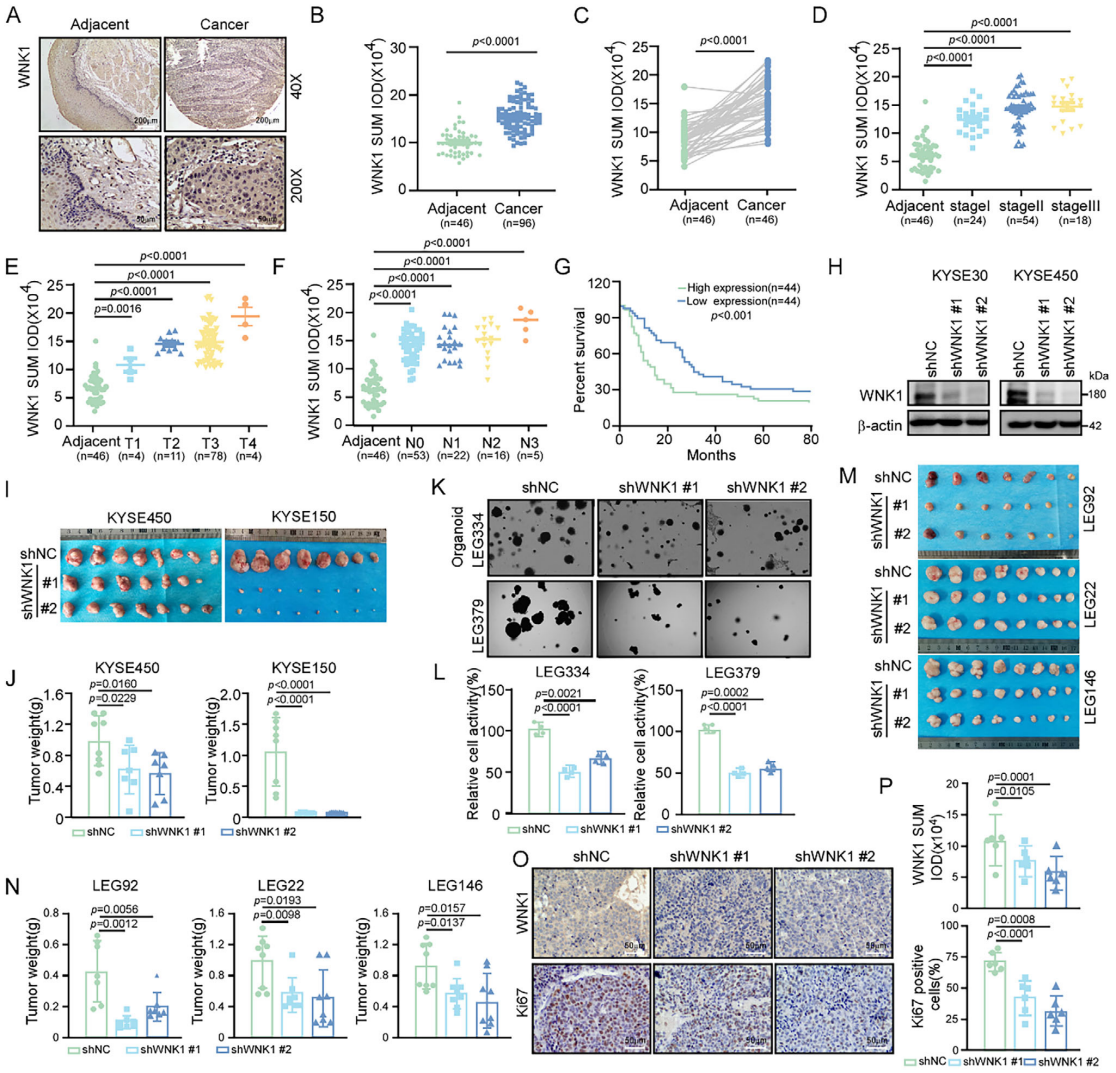
WNK1 is highly expressed in ESCC and negatively correlated with patient prognosis. (A) Representative IHC staining images showing WNK1 levels in ESCC tissues. (B) IHC staining score analysis of unpaired tumoral and adjacent normal tissues for WNK1 expression. (C) WNK1 protein levels in ESCC tissues compared to paired normal tissues by IHC staining score. (D) WNK1 protein levels staining score in varying clinical stages of ESCC. (E) WNK1 protein levels staining score across different pathological tumor grades. (F) WNK1 protein levels staining score in N stages of ESCC. (G) Kaplan‐Meier analysis of overall survival in ESCC patients with high vs. low WNK1 expression. (H) WB analysis of WNK1 knockdown efficiency in ESCC cells after shRNA targeting. (I) CDX tumors were established and treated with intratumoral injection of control or WNK1 shRNA lentivirus. Representative tumors are shown. (J) Quantification of tumor weight in CDX xenografts. (K)Representative images of patient‐derived organoids (PDOs) following WNK1 knockdown (n = 4). (L) Quantification of relative organoid viability following WNK1 knockdown. (M) Representative images of tumor xenografts derived from three PDX models infected with shWNK1 shRNA or control shRNA. Tumors were harvested at endpoint for evaluation (LEG92, n = 7; LEG22 & LEG146, n = 8). (N) Quantification of tumor weights from the PDX models. (O,P) Representative IHC staining and quantitative analysis of WNK1 and Ki67 expression in xenograft tissues. Data are presented as mean ± SD from three independent biological experiments unless otherwise indicated. Statistical significance was determined using two‐tailed unpaired Student's t‐test (B), paired two‐tailed Student's t‐test (C), one‐way ANOVA followed by Tukey's multiple‐comparison test (D–F, J, L, N, and P), and the log‐rank (Mantel‐Cox) test for Kaplan‐Meier survival analysis (G). Exact P values are provided in the Source Data whenever possible.

To further elucidate the biological role of WNK1 in ESCC, we first established stable WNK1 knockdown models in KYSE30 and KYSE450 cells. Efficient depletion of WNK1 protein was confirmed by WB analysis (Figure 3H).

We next systematically evaluated the effects of *WNK1* depletion on ESCC cell proliferation. WB results showed that WNK1 expression was higher in ESCC cell lines than in normal esophageal epithelial cells (Figure S3A). MTT assays demonstrated that WNK1 knockdown significantly suppressed the proliferation of both KYSE30 and KYSE450 cells, which exhibited high endogenous WNK1 expression (Figure S3B). Consistently, colony formation assays showed a marked reduction in clonogenic capacity following WNK1 depletion (Figure S3C,D). Furthermore, EdU incorporation assays revealed a significant decrease in the proportion of EdU‐positive cells after *WNK1* knockdown (Figure S3E,F), indicating impaired DNA synthesis and proliferative activity.

In addition to regulating cell growth, WNK1 also contributed to the migratory and invasive phenotypes of ESCC cells. Wound‐healing assays demonstrated that *WNK1* knockdown markedly delayed wound closure (Figure S3G,H). Consistently, Transwell assays showed that depletion of WNK1 significantly impaired both cell migration (Figure S3I,J) and invasion (Figure S3K,L). Meanwhile, flow cytometric analysis revealed no significant alterations in cell apoptosis following *WNK1* knockdown (Figure S3M,N). These findings indicate that WNK1 promotes ESCC cell migration and invasion independently of apoptosis regulation.

To further validate the oncogenic function of WNK1, WNK1 was ectopically overexpressed in ESCC cells, and successful overexpression was confirmed by WB analysis (Figure S3O). In agreement with the knockdown experiments, WNK1 overexpression significantly enhanced cell proliferation (Figure S3P) and colony‐forming ability (Figure S3Q,R), further supporting a critical role for WNK1 in promoting ESCC cell growth.

We next investigated the role of WNK1 in vivo. In the CDX model, *WNK1* knockdown significantly reduced both tumor volume and tumor weight (Figure 3I,J). To further assess the clinical relevance of these findings, we established patient‐derived organoid (PDO) and PDX models using primary ESCC specimens. *WNK1* depletion markedly inhibited the growth of PDOs (Figure 3K,L) and significantly reduced tumor volume and weight in PDX models (Figure 3M,N), demonstrating that loss of WNK1 suppresses tumor growth in clinically relevant ESCC models. IHC analysis of PDX tumor tissues further showed that *WNK1* knockdown markedly decreased the expression of both WNK1 and the proliferation marker Ki67 (Figure 3O,P), providing additional evidence that WNK1 is required to sustain tumor cell proliferation in vivo.

Collectively, these findings demonstrate that WNK1 functions as a critical promoter of ESCC progression both in vitro and in vivo. Genetic depletion of WNK1 consistently suppresses tumor cell proliferation and tumor growth across cell‐based, organoid, and patient‐derived xenograft models, whereas WNK1 overexpression exerts the opposite effects, establishing WNK1 as an essential driver of ESCC malignant progression.

### 2.4 Knockout of *WNK1* Suppresses 4NQO‐Induced Esophageal Tumor Growth

To investigate the physiological role of WNK1 in ESCC tumorigenesis in vivo, we generated *Wnk1* conditional knockout (*Wnk1*‐cKO) mice using the Cre‐LoxP system by specifically deleting exon 2 of the *Wnk1* gene (Figure 4A). Successful generation of the *Wnk1*‐cKO mice was confirmed by PCR genotyping (Figure 4B). Furthermore, H&E staining and IHC analyses verified efficient depletion of WNK1 expression in the esophageal epithelium of *Wnk1*‐cKO mice (Figure 4C,D).

**FIGURE 4:**
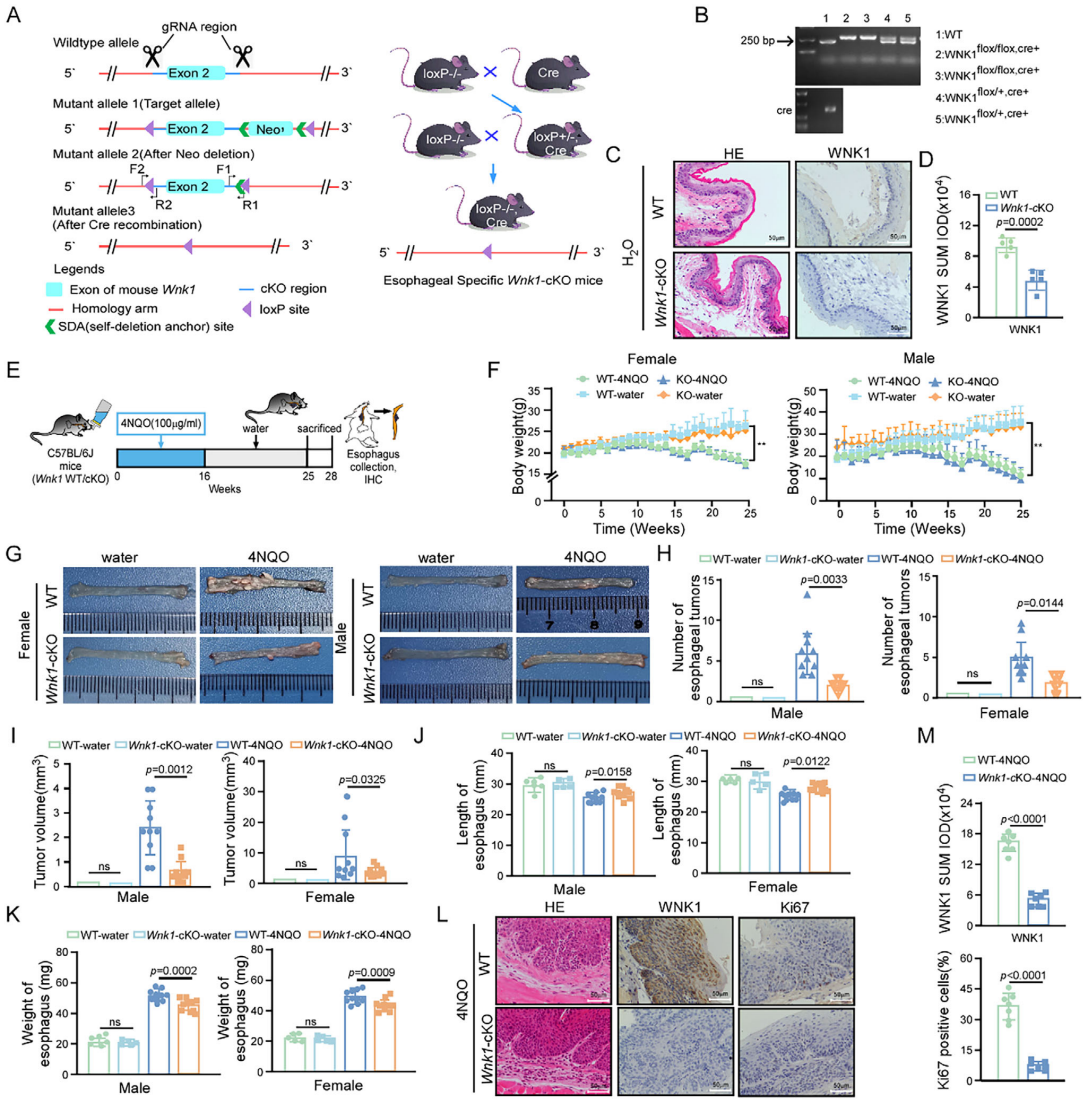
*Wnk1*‐cKO mice dramatically suppress tumor growth in 4NQO‐induced carcinogenic model. (A) Schematic illustrating the generation of esophageal‐specific *Wnk1* conditional knockout (*Wnk1*‐cKO) mice using the Cre‐loxP system. (B) PCR‐based genotyping confirming the *Wnk1* floxed allele and Cre recombinase in WT and *Wnk1*‐cKO mice. (C,D) Representative H&E staining and IHC staining for WNK1 in esophageal tissues from WT and *Wnk1*‐cKO mice, together with quantitative analysis of WNK1 IHC scores. (E) Experimental design of the 4NQO‐induced esophageal carcinogenesis model. (F) Body weight changes of female and male WT and *Wnk1*‐cKO mice during the 4NQO treatment period. (G) Representative images of esophageal tissues harvested from WT and *Wnk1*‐cKO mice (female/male) treated with 4NQO. Arrows indicate carcinomas. (H–K) Quantification of esophageal tumor number (H), tumor volume (I), esophageal length (J), and esophageal weight (K) in female and male WT and *Wnk1*‐cKO mice after 4NQO treatment. (L,M) Representative H&E staining and IHC images of WNK1 and Ki67, together with quantitative analysis of WNK1 expression and Ki67‐positive cells in esophageal tumors from WT and *Wnk1*‐cKO mice. Data are presented as mean ± SD. Statistical significance was determined using Student's t‐test for comparisons between two groups (D and M) and one‐way ANOVA followed by appropriate multiple‐comparison tests for analyses involving more than two groups (F and H‐K). ns, no significant difference; P values are indicated in the figures.

To determine the role of WNK1 in ESCC development, mice were subjected to the well‐established 4‐nitroquinoline 1‐oxide (4NQO)‐induced esophageal carcinogenesis model, which closely recapitulates the pathological progression of human ESCC (Figure 4E). Body weight was monitored throughout the experiment. Following 4NQO treatment, both groups exhibited progressive weight loss, with a marked reduction observed by week 26 compared with water‐treated controls. In contrast, no significant difference in body weight was observed between water‐treated *Wnk1*‐cKO and wild‐type (WT) mice throughout the experimental period (Figure 4F).

At the experimental endpoint, esophageal tissues were harvested for macroscopic and histopathological analyses (Figure 4G). Compared with WT mice, *Wnk1*‐cKO mice developed significantly fewer and smaller esophageal tumors following 4NQO treatment, as evidenced by reduced tumor number (Figure 4H) and tumor size (Figure 4I). These findings demonstrate that genetic ablation of *Wnk1* markedly suppresses 4NQO‐induced ESCC tumorigenesis.

Importantly, under physiological conditions without 4NQO exposure, *Wnk1*‐cKO mice displayed no significant differences in esophageal length, or esophageal weight compared with WT littermates (Figure 4J,K), suggesting that conditional deletion of *Wnk1* does not cause overt alterations in normal esophageal morphology.

To further investigate whether the reduced tumor burden was associated with impaired cellular proliferation, we examined the proliferation marker Ki67 by H&E staining and IHC. Compared with WT mice, *Wnk1*‐cKO mice exhibited markedly reduced WNK1 expression accompanied by a significant decrease in Ki67‐positive cells within the esophageal epithelium (Figure 4L,M), indicating that loss of WNK1 suppresses epithelial cell proliferative activity.

Collectively, these results demonstrate that conditional deletion of Wnk1 significantly inhibits 4NQO‐induced ESCC development while exerting minimal effects on normal esophageal tissue homeostasis, suggesting that WNK1 promotes ESCC tumorigenesis, at least in part, by enhancing the proliferative capacity of esophageal epithelial cells.

### 2.5 WNK1 Promotes ESCC Proliferation by Regulating Histone H3 Ser10 Site Phosphorylation

Having established the oncogenic role of WNK1 in ESCC, we next sought to elucidate the underlying molecular mechanism. To identify proteins associated with WNK1, we performed immunoprecipitation (IP) using an anti‐WNK1 antibody followed by liquid chromatography‐tandem mass spectrometry (LC‐MS/MS) in KYSE30 and KYSE450 cells (Figure 5A). Comparative analysis identified 20 proteins that were commonly enriched in both cell lines. Among these candidates, histone H3 ranked among the top hits based on peptide scores and was therefore selected for further investigation.

**FIGURE 5:**
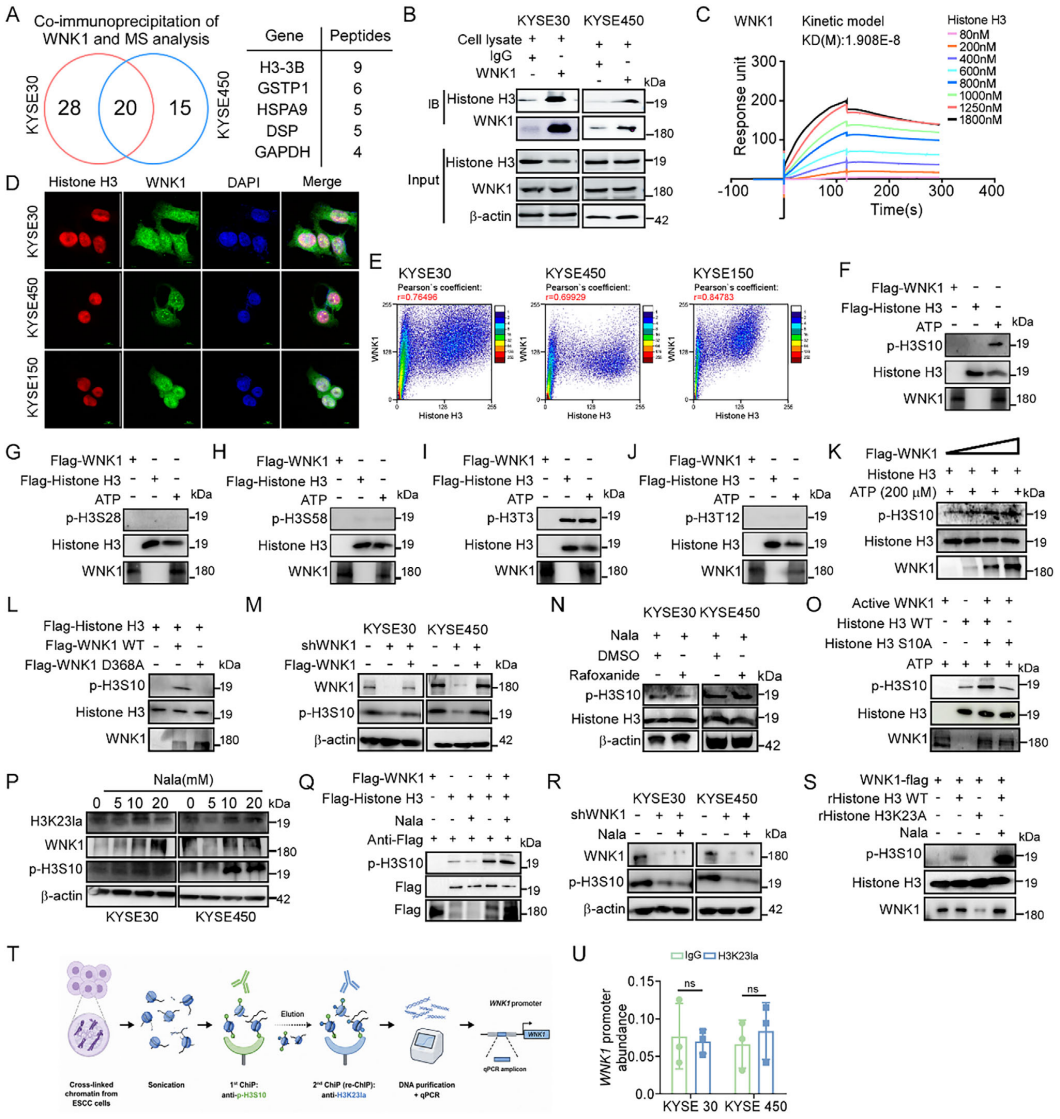
WNK1 phosphorylates histone H3 at Ser10 to promote ESCC proliferation. (A) Venn diagram showing overlapping proteins identified by Co‐IP assay and followed by MS analysis in ESCC cells. (B) Co‐IP analysis confirming interaction between WNK1 and histone H3 in ESCC cells. (C) SPR analysis of the WNK1‐histone H3 interaction. (D,E) IF staining demonstrated the spatial colocalization of histone H3 and WNK1, and Pearson's correlation analysis was performed. (F–J) In vitro kinase assay showing WNK1‐mediated phosphorylation of histone H3 at different sites in the presence of ATP. (K) In vitro kinase assay showing WNK1‐mediated phosphorylation of histone H3 at Ser10 in an WNK1‐dependent manner. (L) In vitro kinase assay comparing wild‐type WNK1 (WT) and the kinase‐dead mutant WNK1‐D368A. The D368A mutant failed to phosphorylate histone H3 at Ser10. (M) WB analysis showing that WNK1 knockdown reduces p‐H3S10 expression, whereas ectopic expression of Flag‐WNK1 restores p‐H3S10 levels in KYSE30 and KYSE450 cells. (N) WB analysis showing that treatment with the SPAK/OSR1 inhibitor rafoxanide (5 µM) did not reduce p‐H3S10 levels in KYSE30 and KYSE450 cells. (O) In vitro kinase assay using histone H3 WT or mutant histone H3 S10A as substrates. (P) WB analysis showing that treatment with NaLa increased the level of H3K23la, WNK1, and p‐H3S10 in KYSE30 and KYSE450 cells. (Q) IP experiment was performed after treatment with NaLa. (R) WB analysis examined p‐H3S10 expression after WNK1 knockdown and subsequent Nala treatment. (S) WB analysis was used to detect the effect of intracellular recombinant wild‐type histone H3 (rHistone H3 WT) or H3K23A mutant (rHistone H3 K23A) on p‐H3S10 expression. (T) Schematic diagram of ChIP‐re‐ChIP. (U) ChIP‐re‐ChIP experiment was performed and analyzed. Data are presented as mean ± SD from three independent experiments unless otherwise indicated. Statistical significance was determined using Student's t‐test.

Endogenous co‐immunoprecipitation (Co‐IP) confirmed the association between WNK1 and histone H3 in both KYSE30 and KYSE450 cells (Figure 5B). Surface plasmon resonance (SPR) analysis further demonstrated a strong binding affinity between the two proteins, with a dissociation constant (KD) of 1.908 × 10^−^^8^ M (Figure 5C). Consistently, immunofluorescence analysis revealed substantial nuclear co‐localization of WNK1 and histone H3 (Figure 5D,E). Together, these results indicate that WNK1 physically associates with histone H3 in the nucleus.

Given that WNK1 is a serine/threonine kinase, we next investigated whether histone H3 serves as a direct substrate of WNK1. An in vitro kinase assay using recombinant histone H3 identified robust phosphorylation at Ser10, whereas no obvious phosphorylation was detected at other reported H3 phosphorylation sites, including Ser28, Ser58, Thr3, and Thr12 (Figure 5F–J). Moreover, increasing concentrations of recombinant WNK1 produced a dose‐dependent elevation of H3 Ser10 phosphorylation in vitro (Figure 5K). These results suggest that WNK1 can phosphorylate unmodified recombinant histone H3 at Ser10 in vitro, indicating that pre‐existing H3K23 lactylation is not biochemically required for WNK1 catalytic activity toward H3S10. Importantly, the kinase‐dead mutant WNK1‐D368A failed to induce H3 Ser10 phosphorylation (Figure 5L), indicating that WNK1 kinase activity is required for H3 Ser10 phosphorylation. Manipulating WNK1 expression in ESCC cells further demonstrated that p‐H3S10 levels positively correlated with WNK1 abundance, as WNK1 knockdown markedly decreased, whereas WNK1 overexpression increased, H3 Ser10 phosphorylation (Figure 5M). To determine whether the WNK1‐associated regulation of p‐H3S10 was independent of cell‐cycle alterations, cells were analyzed by flow cytometry using PI staining together with p‐H3S10 staining to identify mitotic cells. WNK1 knockdown did not significantly alter the proportions of cells in the G1, S, G2, or M phases compared with control cells (Figure S4A–C). Similarly, re‐expression of WNK1‐WT or the kinase‐deficient D368A mutant in WNK1‐depleted cells did not markedly affect cell‐cycle distribution (Figure S4D–H). These findings indicate that the changes in p‐H3S10 associated with WNK1 expression are unlikely to result from altered cell‐cycle distribution, supporting a direct regulatory effect of WNK1 on H3S10 phosphorylation. To exclude the possibility that H3 Ser10 phosphorylation was mediated through the canonical WNK1‐SPAK/OSR1 signaling cascade, cells were treated with a selective SPAK/OSR1 inhibitor. Inhibition of SPAK/OSR1 had no detectable effect on p‐H3S10 levels (Figure 5N), suggesting that WNK1 regulates H3 Ser10 phosphorylation independently of its classical downstream signaling pathway. Collectively, these findings demonstrate that histone H3 is a direct substrate of WNK1 and that Ser10 is its major phosphorylation site.

To further verify the phosphorylation site, we generated phospho‐deficient (S10A) and phospho‐mimetic (S10D) mutants of histone H3. In vitro kinase assays showed that WNK1 efficiently phosphorylated wild‐type H3 but completely failed to phosphorylate the S10A mutant (Figure 5O), confirming that Ser10 is the specific residue targeted by WNK1. To determine the functional significance of H3S10 phosphorylation in ESCC, we performed rescue experiments using wild‐type H3 (H3‐WT), the phospho‐mimetic H3‐S10D mutant, and the non‐phosphorylatable H3‐S10A mutant. In H3‐depleted cells, re‐expression of H3‐WT or H3‐S10D restored cell proliferation and colony‐forming capacity, whereas H3‐S10A failed to rescue these effects (Figure S5A–C). Notably, in WNK1‐depleted cells, H3‐S10D, but not H3‐WT or H3‐S10A, substantially restored cell proliferation and colony formation (Figure S5D–F), supporting H3S10 phosphorylation as a downstream effector of WNK1. Similarly, H3‐S10D rescued the proliferative defects induced by 2‐DG treatment, whereas H3‐WT and H3‐S10A showed limited or no rescue effects (Figure S5G–I). Together, these findings support a functional link between glycolytic activity, WNK1, and H3S10 phosphorylation in promoting ESCC cell growth.

To further investigate the role of H3K23 lactylation in the regulation of H3S10 phosphorylation through WNK1, ESCC cells were treated with NaLa to enhance H3K23 lactylation. NaLa treatment markedly increased H3K23la levels, accompanied by increased WNK1 expression and H3S10 phosphorylation (Figure 5P). These findings further support a model in which H3K23 lactylation promotes H3S10 phosphorylation through upregulation of WNK1 expression. Because our previous findings suggested that lactate activates WNK1, we next investigated whether lactate regulates H3 Ser10 phosphorylation. IP results showed that treatment with NaLa (10 mM) markedly enhanced WNK1‐mediated H3 Ser10 phosphorylation in cells (Figure 5Q). Furthermore, WB analysis showed that WNK1 knockdown reduced p‐H3S10 expression, and the addition of NaLa (10 mM) did not alleviate the p‐H3S10 downregulation caused by WNK1 knockdown (Figure 5R). To determine whether H3K23la regulates H3S10 phosphorylation through WNK1 expression, endogenous H3 was depleted and replaced with WT H3 or the lactylation‐deficient H3K23A mutant. Compared with WT H3, H3K23A reduced WNK1 and p‐H3S10 levels, whereas NaLa (10 mM) markedly increased H3S10 phosphorylation in WT H3‐reconstituted cells (Figure 5S). These findings indicate that H3K23la enhances H3S10 phosphorylation by promoting WNK1 expression.

To further determine whether H3K23la regulates p‐H3S10 through a trans‐acting mechanism, namely by promoting WNK1 expression and thereby indirectly modulating global p‐H3S10, or through cis crosstalk between H3K23la and p‐H3S10, we performed a sequential ChIP (ChIP‐re‐ChIP) assay. Chromatin was first immunoprecipitated with an anti‐p‐H3S10 antibody, followed by elution and a second round of immunoprecipitation with an anti‐H3K23la antibody. The resulting chromatin was subsequently analyzed by qPCR targeting the *WNK1* promoter region. Conventional ChIP‐qPCR confirmed significant enrichment of H3K23la at the *WNK1* promoter. However, sequential p‐H3S10→H3K23la ChIP‐re‐ChIP analysis revealed no significant co‐enrichment of p‐H3S10 and H3K23la at the examined *WNK1* promoter regions (Figure 5T,U). These findings demonstrate that H3K23la directly occupies the WNK1 promoter without forming a detectable cis regulatory complex with p‐H3S10. Instead, H3K23la promotes WNK1 transcription, which subsequently mediates H3S10 phosphorylation through a trans‐acting mechanism.

Given that histone H3 phosphorylation occurs in the nucleus, we next investigated whether lactate regulates the subcellular localization of WNK1 to facilitate its nuclear function. WB analysis following nuclear‐cytoplasmic fractionation showed that treatment with 2‐DG (1 mM) markedly reduced the nuclear abundance of WNK1, whereas NaLa (10 mM) treatment significantly increased nuclear WNK1 levels (Figure S5J). Consistently, immunofluorescence (IF) analysis further demonstrated that 2‐DG treatment decreased the nuclear localization of WNK1, whereas NaLa promoted its nuclear accumulation (Figure S5K). Quantification of the nuclear‐to‐cytoplasmic (N/C) ratio of WNK1 further confirmed that 2‐DG significantly decreased the N/C ratio of WNK1, while NaLa significantly increased it (Figure S5L). Collectively, these findings indicate that lactate availability is closely associated with the nuclear accumulation of WNK1 and further support a role for lactate in promoting WNK1‐mediated histone H3 phosphorylation.

Furthermore, Co‐IP analysis revealed an interaction between WNK1 and Importin α5 (Figure S5M). We next analyzed the WNK1 protein sequence using the cNLS Mapper online tool (https://nls‐mapper.iab.keio.ac.jp/cgi‐bin/NLS_Mapper_y.cgi) and identified a putative classical nuclear localization signal (NLS)‐like motif spanning amino acids 563–572 of WNK1. To further investigate whether the nuclear import of WNK1 is dependent on the importin machinery, ESCC cells were treated with NaLa to promote the nuclear accumulation of WNK1, followed by treatment with the importin inhibitor ivermectin (IVM; 10 µM) for 24 h. IF analysis showed that NaLa treatment markedly increased the nuclear abundance of WNK1, whereas IVM substantially attenuated NaLa‐induced WNK1 nuclear accumulation (Figure S5N,O). Collectively, these findings support an importin‐dependent mechanism for WNK1 nuclear import and suggest that Importin α5 may facilitate WNK1 nuclear entry through recognition of its putative NLS‐like motif, thereby contributing to lactate‐induced nuclear accumulation of WNK1.

Collectively, these findings identify histone H3 as a previously unrecognized substrate of WNK1. Upon lactate stimulation, WNK1 accumulates in the nucleus and directly phosphorylates histone H3 at Ser10 in an H3K23 lactylation‐dependent manner, thereby promoting ESCC cell proliferation. These results uncover a previously unappreciated epigenetic function of WNK1 and establish the H3K23la–WNK1–H3S10 signaling axis as a key mechanism driving ESCC progression.

### 2.6 p‐H3S10 Activates AKT1 Transcription in ESCC

Previous studies have demonstrated that H3K23la binds to the promoter region of *WNK1* and enhances its transcriptional activity. Based on this finding, we hypothesized that lactate metabolism‐induced H3K23la upregulates WNK1 expression, thereby increasing p‐H3S10 level, activating downstream oncogenic signaling, and ultimately driving ESCC progression.

To further test this hypothesis, ESCC cells were treated with the glycolysis inhibitor DCA, Oxamate, 2‐DG to modulate intracellular lactate levels. WB analysis revealed that glycolysis inhibitor treatment markedly reduced the expression of H3K23la, WNK1, and p‐H3S10 (Figure S6A). These findings indicate that lactate metabolism promotes WNK1 expression through regulation of H3K23la, thereby enhancing p‐H3S10 levels.

To determine the clinical relevance of these observations, we examined the expression of H3K23la, WNK1, and p‐H3S10 in primary ESCC tissues and their matched adjacent normal tissues. The patient inclusion and cohort allocation workflow is summarized in Figure S6B. The initial ESCC tissue microarray cohort included tumor tissues and matched adjacent non‐tumor tissues. Following IHC staining and quality assessment, all evaluable samples were included in the non‐paired analysis, whereas matched tumor–adjacent tissue pairs were used for paired comparisons. In addition, patients with evaluable tumor IHC scores and complete follow‐up data were included in the Kaplan–Meier survival analysis and stratified into high‐ and low‐expression groups according to the predefined cutoff. H3K23la, WNK1, and p‐H3S10 were significantly upregulated in tumor tissues compared with adjacent normal tissues, and H3K23la expression positively correlated with p‐H3S10 levels (Figure 6A,B). IHC analysis of a commercial ESCC tissue microarray further demonstrated that p‐H3S10 expression was markedly elevated in tumor tissues relative to adjacent non‐tumorous tissues (Figure 6C–E). Moreover, elevated p‐H3S10 expression was significantly associated with higher histological grade, advanced TNM stage, deeper tumor invasion (T3‐T4), and poorer overall survival (Figure 6F–I). Consistently, tissue microarray analysis confirmed a significant positive correlation between H3K23la and p‐H3S10 expression (Figure 6J), supporting the clinical relevance of the H3K23la–WNK1–p‐H3S10 signaling axis in ESCC progression.

**FIGURE 6:**
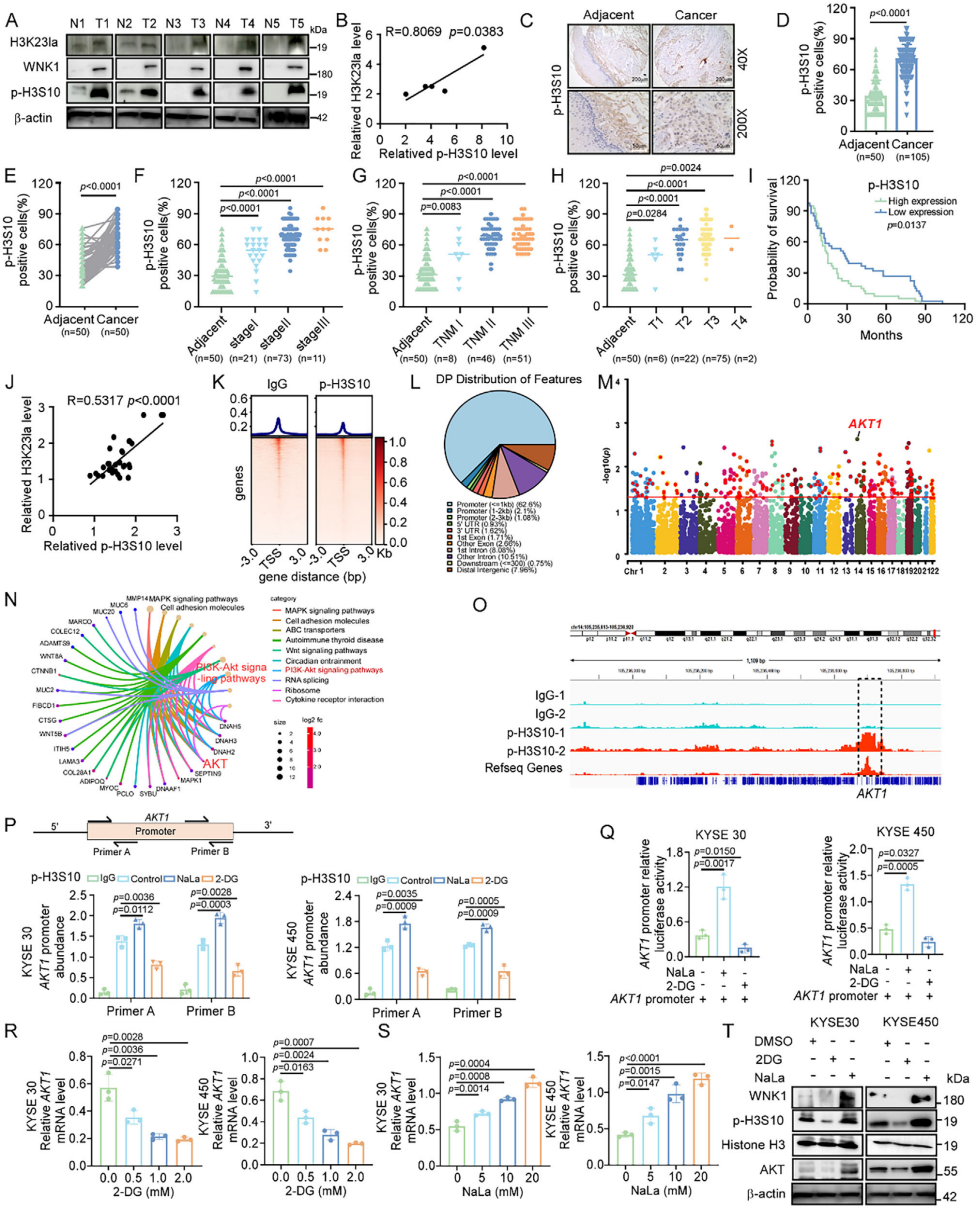
Expression profiling and regulatory relevance of p‐H3S10 and H3K23la in ESCC. (A) WB analysis of WNK1, H3K23la, and p‐H3S10 protein levels in paired ESCC tumors and adjacent normal tissues. (B) The correlation between H3K23la and p‐H3S10 expression was analyzed using Pearson correlation analysis. (C–E) Representative IHC staining for p‐H3S10 in adjacent normal tissues and ESCC tissues (C), together with quantitative analyses of p‐H3S10 expression in unpaired (D) and paired (E) clinical cohorts. (F) Quantification of p‐H3S10 expression according to pathological stage. (G,H) Quantification of p‐H3S10 expression in adjacent tissues and tumors of different pathological stages, TNM stages and T classifications. (I) Kaplan‐Meier analysis of overall survival in ESCC patients with high vs. low p‐H3S10 expression. (J) Pearson correlation analysis between H3K23la and p‐H3S10 protein expression levels across ESCC clinical specimens. Each dot represents one tissue sample. R and *P* values are indicated. (K) Genome‐wide distribution of p‐H3S10 CUT‐Tag peaks across genomic features. (L) Genomic annotation of p‐H3S10 peaks showing the distribution of promoter and non‐promoter regions. (M) Manhattan plot showing p‐H3S10‐enriched chromatin regions across chromosomes. (N) Top 10 KEGG pathway‐associated DEGs related to the p‐H3S10 enrichment regions. DEGs are shown in a color gradient based on their log2fold change (FC) values. (O) WashU Epigenome Browser tracks showing p‐H3S10 (red) signals of representative upregulated *AKT1* genes in ESCC cells, each with two replicates. The p‐H3S10 GAIN regions are shaded in red. (P) ChIP‐qPCR analysis showing p‐H3S10 enrichment at two regions (Primer A and Primer B) within the *AKT1* promoter following NaLa or 2‐DG treatment (n = 3). (Q) Dual‐luciferase reporter assays showing *AKT1* promoter activity following NaLa (10 mM) or 2‐DG (1 mM) treatment (n = 3). (R,S) qRT‐PCR analysis of *AKT1* mRNA levels after 2‐DG or NaLa treatment at indicated concentrations in ESCC cells (n = 3). (T) WB analysis of WNK1, p‐H3S10, and AKT expression following NaLa or 2‐DG treatment. Data are presented as mean ± SD from three independent experiments unless otherwise indicated. Statistical significance was determined using Student's t‐test for two‐group comparisons and one‐way ANOVA followed by appropriate multiple‐comparison tests for multiple‐group comparisons. ns, no significant difference.

Given that H3 Ser10 phosphorylation is a well‐established chromatin modification involved in transcriptional activation, we next investigated its downstream regulatory mechanism [40]. CUT‐Tag sequencing in KYSE450 cells revealed that p‐H3S10 was predominantly enriched at promoter regions and was concentrated around transcription start sites (TSSs), suggesting a role in transcriptional regulation (Figure 6K,L). Notably, one of the strongest enrichment peaks was identified at the *AKT1* promoter (Figure 6M). Integration of CUT‐Tag data with RNA‐seq‐derived differentially expressed genes followed by KEGG pathway enrichment analysis identified the PI3K‐AKT signaling pathway as one of the most significantly enriched pathways (Figure 6N). Genome browser visualization further confirmed a robust and specific p‐H3S10 enrichment peak at the *AKT1* promoter (Figure 6O), suggesting that AKT is a direct transcriptional target of p‐H3S10.

To validate this mechanism, we performed ChIP‐qPCR and the result demonstrated that NaLa treatment significantly increased p‐H3S10 occupancy at the *AKT1* promoter, whereas 2‐DG markedly reduced its enrichment (Figure 6P), indicating that lactate metabolism regulates p‐H3S10 enrichment at the *AKT1* promoter. In contrast, H3K23la enrichment at the *AKT1* promoter remained unchanged under either treatment (Figure S6C), suggesting that AKT1 is not directly regulated by H3K23la but rather through p‐H3S10‐mediated transcriptional activation. Consistently, dual‐luciferase reporter assays showed that NaLa significantly enhanced *AKT1* promoter activity, whereas 2‐DG markedly suppressed it (Figure 6Q). RT‐qPCR further demonstrated that 2‐DG reduced *AKT1* mRNA expression in a dose‐dependent manner, while NaLa significantly increased AKT transcription (Figure 6R,S). WB analysis confirmed that 2‐DG treatment decreased the protein levels of WNK1, p‐H3S10, AKT, and p‐AKT(S473), p‐mTOR(S2448), whereas NaLa treatment exerted the opposite effects (Figure 6T and Figure S6D). Furthermore, correlation analysis also showed significant positive association between WNK1 and AKT1 mRNA expression in ESCC (Figure S6E). Collectively, these findings demonstrate that lactate metabolism activates the AKT/mTOR signaling pathway through the H3K23la–WNK1–p‐H3S10 signaling axis.

To further establish the essential role of WNK1 in this signaling cascade, we performed both in vitro and in vivo validation. WNK1 knockdown markedly reduced the expression of p‐H3S10 and p‐AKT(S473) (Figure S6F,G). Similarly, in the 4NQO‐induced ESCC mouse model, esophageal tissues from *Wnk1*‐cKO mice exhibited substantially lower levels of p‐H3S10 and p‐AKT(S473) than those from wild‐type mice (Figure S6H,I), further confirming that WNK1 functions upstream of AKT signaling. Analysis of public datasets further demonstrated that high WNK1 expression and elevated PI3K‐AKT pathway activity were both significantly associated with poor overall survival in patients with ESCC (Figure S6J,K). Furthermore, treatment with the AKT inhibitor MK‐2206 failed to alter the expression of WNK1 or p‐H3S10 (Figure S6L), indicating that AKT does not exert feedback regulation on the WNK1–p‐H3S10 signaling axis. Given that p300/CBP has been reported to possess histone lactyltransferase activity, we further investigated whether p300/CBP contributes to lactate‐induced WNK1 expression and downstream signaling activation. Pharmacological inhibition of p300/CBP activity using A‐485 markedly reduced H3K23la levels in KYSE30 and KYSE450 cells in a dose‐dependent manner. Consistently, A‐485 treatment decreased WNK1 expression, p‐H3S10 and p‐AKT(S473) levels (Figure S6M), suggesting that p300/CBP activity is involved in H3K23 lactylation and the subsequent activation of the WNK1–p‐H3S10–AKT signaling axis.Because our study focuses on lactate‐associated metabolic reprogramming, we first sought to identify the cellular compartment most likely responsible for enhanced glycolysis and lactate production within the ESCC tumor microenvironment. We therefore examined LDHA expression across the major annotated malignant, immune, and stromal cell populations. The scRNA‐seq analysis showed that LDHA expression was predominantly enriched in malignant squamous epithelial cells (Figure S7A–C), indicating that the malignant epithelial compartment represents the principal LDHA‐high, glycolytically active cell population in ESCC. Stratification according to LDHA expression further revealed that LDHA‐high tumors contained a lower proportion of T cells, whereas LDHA‐low tumors exhibited increased T‐cell abundance (Figure S7D,E). Consistent with these findings, immune infiltration analysis showed significantly higher adaptive immune‐cell infiltration, particularly B cells and CD8^+^ T cells, in the LDHA‐low group (Figure S7F), suggesting an inverse association between LDHA expression and anti‐tumor immune infiltration. In addition, WNK1 and AKT1 transcripts were preferentially expressed in malignant epithelial cells (Figure S7G,H), supporting the cellular localization of the proposed LDHA–H3K23la–WNK1 signaling axis.

To further validate these findings in situ, we performed multiplex immunofluorescence (mIF) analysis to simultaneously examine the spatial distribution of key components of the proposed signaling pathway and the associated tumor microenvironment. Specifically, Pan‐CK was used to identify malignant epithelial cells, LDHA to indicate glycolytic/lactate metabolic activity, H3K23la to assess histone lactylation, WNK1, p‐H3S10, and p‐AKT(S473) to evaluate activation of the proposed LDHA–H3K23la–WNK1–p‐H3S10–AKT signaling cascade, whereas FAP and CD8α were used to assess cancer‐associated fibroblast (CAF) enrichment and cytotoxic T‐cell infiltration, respectively.

Compared with adjacent Pan‐CK‐negative regions, Pan‐CK‐positive tumor regions exhibited significantly higher expression of LDHA, H3K23la, WNK1, p‐H3S10, and p‐AKT(S473) (Figure S7I–N). These markers showed concordant spatial enrichment within tumor regions, supporting coordinated activation of the proposed signaling pathway in situ. Furthermore, regions enriched for these pathway‐associated markers exhibited significantly increased FAP expression and markedly reduced CD8α^+^ T‐cell infiltration (Figure S7O,P), demonstrating a spatial association between activation of the LDHA–H3K23la–WNK1–p‐H3S10–AKT pathway, CAF enrichment, and an immunosuppressive tumor microenvironment. These findings suggest that, in addition to activating the H3K23la–WNK1–p‐H3S10–AKT signaling axis in tumor cells, lactate accumulation may further promote ESCC progression by establishing an immunosuppressive tumor microenvironment.

Taken together, our finding defines a lactate–H3K23la–WNK1–p‐H3S10–AKT axis connecting metabolism to oncogenic signaling. Concurrently, lactate metabolism contributes to the establishment of an immunosuppressive tumor microenvironment, collectively driving ESCC progression. These findings establish the “Lactate–H3k23la–Wnk1–P‐H3s10–Akt” signaling axis as a mechanistic link between metabolic reprogramming and epigenetic regulation in ESCC and provide potential therapeutic targets for intervention.

### 2.7 CD03017 can Inhibit the Growth of ESCC In Vitro and In Vivo

To identify potential WNK1 inhibitors, we used AutoDock Vina software to perform molecular docking screening on 53,344 small molecules from the Maybridge database. Based on the screening results, the six small molecules with the highest docking affinities were identified (Figure S8A). The docking models and structural formulas of these six compounds bound to the WNK1 protein are shown (Figure S8B). MTT assays were then used to evaluate the effects of the potential WNK1 inhibitors on ESCC cell proliferation. The results showed that CD03017 exhibited the greatest inhibitory effect on ESCC proliferation (Figure S8C). Therefore, CD03017 (structure shown in Figure 7A) was selected for subsequent studies.

**FIGURE 7:**
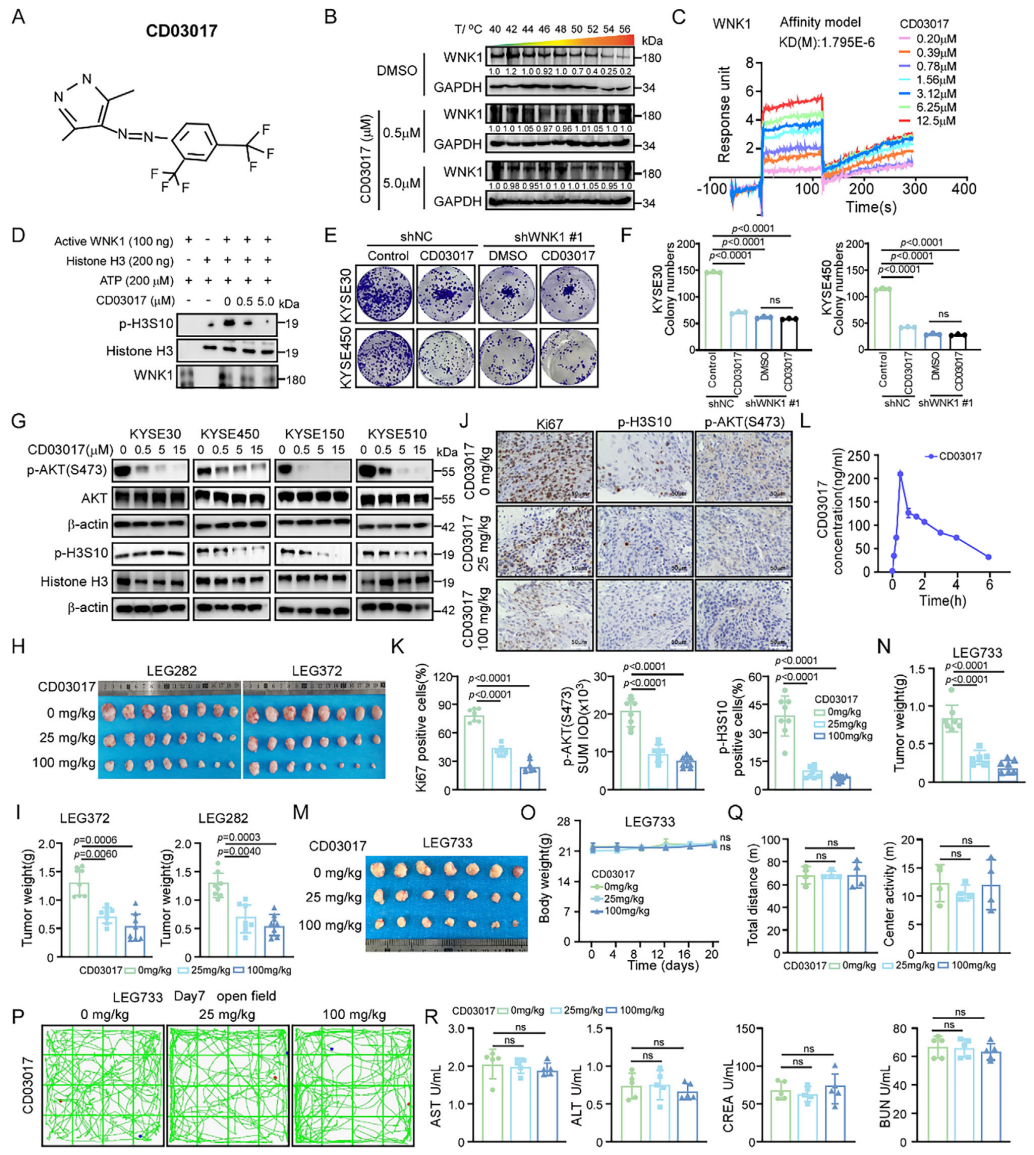
CD03017 selectively targets WNK1 kinase activity and suppresses ESCC growth in vitro and in vivo. (A) Chemical structure of CD03017. (B) CETSA assay was performed to check the interaction between WNK1 and CD03017. (C) SPR experiment analysis of the WNK1‐CD03017 interaction. (D) In vitro kinase assay showing that CD03017 dose‐dependently inhibited WNK1‐catalyzed phosphorylation of histone H3 at Ser10. (E,F) Colony formation assays and corresponding quantification showing that CD03017 markedly suppressed clonogenic growth of KYSE30 and KYSE450 cells. Silencing WNK1 largely abolished the additional inhibitory effect of CD03017, indicating that its antitumor activity is primarily mediated through WNK1. (G) WB analysis showing that CD03017 dose‐dependently reduced phosphorylation of H3S10 and AKT (S473). (H) Representative images of PDX tumors following treatment with CD03017. (I) Tumor weight analysis of LEG372 and LEG282 PDX tumors showing that CD03017 treatment significantly reduced tumor burden (n = 7). (J,K) Representative IHC staining and quantitative analysis of Ki67, p‐H3S10 and p‐AKT(S473) (n = 8). (L) Plasma concentration–time profile of CD03017 in mice determined by HPLC analysis (n = 3). (M,N) Representative images and quantification of LEG733 PDX tumors demonstrating dose‐dependent suppression of tumor growth by CD03017. (O) Body weight changes of mice bearing LEG733 PDX tumors during treatment with the indicated doses of CD03017, showing no significant differences among groups. (P,Q) Representative open‐field tracking images and quantification of total distance traveled and center activity, indicating that CD03017 did not affect locomotor activity or exploratory behavior (n = 4). (R) Serum biochemical analyses showing comparable AST, ALT, CREA, and BUN levels among treatment groups, indicating no apparent hepatic or renal toxicity (n = 5). Data are presented as mean ± SD from three independent experiments unless otherwise indicated. Statistical significance was determined using one‐way ANOVA followed by multiple‐comparison tests. ns, no significant difference.

To determine whether CD03017 directly binds to WNK1, we first performed a thermal shift assay. CD03017 markedly increased the thermal stability of WNK1 over the temperature range of 40°C–56 °C (Figure 7B), suggesting a direct interaction between the compound and the protein. This interaction was further confirmed by the cellular thermal shift assay (CETSA). SPR analysis further demonstrated a strong binding affinity between CD03017 and WNK1, with a KD of 1.795 × 10^−^^6^ M (Figure 7C). Next, we investigated whether CD03017 inhibits the kinase activity of WNK1. An in vitro kinase assay showed that CD03017 dose‐dependently suppressed WNK1‐mediated p‐H3S10 (Figure 7D), indicating that CD03017 directly inhibits WNK1 kinase activity. To further verify that WNK1 is the primary functional target of CD03017, WNK1‐knockdown cells were treated with the compound. CD03017 markedly inhibited colony formation in control cells, whereas its inhibitory effect was substantially attenuated following WNK1 depletion (Figure 7E,F). To further validate the target specificity of CD03017 toward WNK1, a kinase profiling assay was performed. The results demonstrated that CD03017 selectively inhibited WNK1, with no detectable inhibitory activity against other members of the WNK kinase family (Table S2). These findings indicate that the antitumor activity of CD03017 is primarily dependent on WNK1, further supporting WNK1 as its principal molecular target.

We next evaluated the biological effects of CD03017 in ESCC cells. WB analysis revealed that CD03017 dose‐dependently reduced the expression of p‐H3S10 and its downstream effector p‐AKT (Figure 7G), indicating effective inhibition of the WNK1–p‐H3S10–AKT signaling axis. Consistently, MTT assays demonstrated that CD03017 suppressed ESCC cell proliferation in both a dose‐ and time‐dependent manner (Figure S9A). Furthermore, CD03017 markedly reduced colony formation in crystal violet assays (Figure S9B,C), and significantly impaired anchorage‐independent growth in soft agar assays (Figure S9D,E), all the results confirming its potent antiproliferative activity.

To determine the in vivo antitumor efficacy of CD03017, we evaluated the compound in two independent PDX models of ESCC. Intraperitoneal administration of CD03017 (25 or 100 mg/kg) significantly inhibited tumor growth and reduced tumor weight in a dose‐dependent manner (Figure 7H,I). IHC analysis further showed markedly decreased expression of Ki67, p‐H3S10, and p‐AKT in tumors from CD03017‐treated mice (Figure 7J,K), indicating effective suppression of WNK1 downstream signaling in vivo. Given the robust antitumor activity of CD03017 in vivo, we next characterized its pharmacokinetic profile to assess systemic exposure and disposition and to further evaluate its potential for in vivo therapeutic application. High‐performance liquid chromatography (HPLC) analysis showed that CD03017 exhibited an elimination half‐life (t_1_/_2_) of 1.633 h, with its plasma concentration declining rapidly after administration and reaching a low level within 6 h. The area under the concentration‐time curve (AUC) was 532.9 ng·h/mL, and the clearance (CL) was 18.8 L/h/kg (Figure 7L). Together, these findings demonstrate that CD03017 exerts robust antitumor activity in ESCC PDX models despite relatively rapid systemic elimination, supporting its therapeutic potential while providing a pharmacokinetic basis for further optimization of dosing strategies.

To further evaluate the in vivo antitumor activity and safety profile of CD03017, we validated its effects in an independent PDX model. CD03017 treatment consistently suppressed tumor growth (Figure 7M,N). Meanwhile, no significant changes in body weight were observed throughout the treatment period (Figure 7O), and behavioral assessments revealed no apparent abnormalities (Figure 7P,Q). Furthermore, histopathological examination of the liver and kidney showed no obvious treatment‐related tissue damage or toxic effects (Figure 7R), further confirming that CD03017 has good safety in vivo. Collectively, these results demonstrate that CD03017 exerts potent antitumor activity and good in vivo safety in PDX models and exhibits a well‐defined pharmacokinetic profile, supporting its further preclinical development.

Finally, we investigated whether the H3K23la–WNK1–p‐H3S10–AKT signaling axis exerts feedback regulation on glycolysis. Treatment with the AKT inhibitor MK‐2206 did not significantly alter lactate production (Figure S9F). Consistently, Seahorse extracellular flux analysis showed that neither the WNK1 inhibitor CD03017 nor the AKT inhibitor MK‐2206 significantly affected the extracellular acidification rate (ECAR) (Figure S9G–L). These findings indicate that inhibition of WNK1 or its downstream AKT signaling does not impair glycolytic activity, suggesting that the H3K23la–WNK1–p‐H3S10–AKT pathway functions predominantly as a unidirectional signaling cascade downstream of glycolysis, with little or no feedback regulation on glycolytic metabolism.

## 3 Discussion

ESCC remains a highly aggressive malignancy with limited targeted therapeutic options, highlighting a critical need to define actionable molecular mechanisms. Although the Warburg effect has long been recognized [41, 42, 43], how lactate accumulation mechanistically drives oncogenic signaling through epigenetic regulation remains incompletely understood [44, 45]. In this study, we address this gap by identifying a previously unrecognized lactate–H3K23la–WNK1–p‐H3S10–AKT axis that functionally links metabolic reprogramming to chromatin remodeling and tumor progression.

Histone lactylation has emerged as a key mechanism connecting metabolism to gene regulation since its initial description by Zhang et al. [20]. Consistent with this paradigm, we found that H3K23la is markedly upregulated in ESCC and correlates with poor clinical outcomes, supporting its role as both a functional epigenetic regulator and a potential prognostic biomarker. Mechanistically, H3K23la promotes WNK1 transcription, establishing a direct link between lactate accumulation and oncogenic signaling. Our results further suggest that p300 contributes to H3K23la deposition, as pharmacological inhibition of p300 reduced both H3K23la and WNK1 expression. However, whether additional lactyltransferases participate in this process remains to be determined.

Histone lactylation has been reported at multiple lysine residues [20], Notably, previous epigenetic studies have suggested that lysine residues located in the central region of the H3 N‐terminal tail, particularly K18 and K23, participate in regulatory crosstalk with transcription‐activating histone marks. For example, acetylation at H3K18 and H3K23 has been shown to stimulate MLL‐mediated H3K4 methylation, a hallmark of active transcription, indicating that modifications at these residues are closely linked to transcriptional activation and chromatin remodeling [46, 47]. In addition, the MORF histone acetyltransferase complex has been shown to specifically acetylate H3K23 and colocalize with active chromatin marks at target genes, further supporting the regulatory role of this region in transcriptional control [48]. Jiang et al. found that PDHX acetylation promotes tumor progression by disrupting PDC assembly and activating lactate‐mediated gene expression, thereby linking acetylation and histone lactylation in the progression of hepatocellular carcinoma [49]. The functional significance of individual sites remains context‐dependent. Our findings highlight H3K23la as a critical regulatory node in ESCC, extending previous studies largely focused on H3K18la [21, 26, 50, 51, 52]. These results suggest that distinct lactylation sites may exert nonredundant roles in transcriptional regulation and tumor progression.

A key conceptual advance of this study is the identification of WNK1 as a chromatin‐regulating kinase [53]. Although WNK1 has been implicated in cancer progression [29, 30, 31, 32], its role in histone modification has not been previously described. We demonstrate for the first time that WNK1 directly phosphorylates histone H3 at serine 10 site. Moreover, WNK1‐mediated p‐H3S10 is enriched at oncogenic promoters such as AKT, thereby linking epigenetic modification to activation of the PI3K–AKT pathway, a central driver of tumor growth and survival [54, 55, 56, 57, 58, 59].

Lactate accumulation is a characteristic metabolic feature of the tumor microenvironment and is also evident in ESCC. Both L‐ and D‐lactate levels have been reported to be significantly elevated in human ESCC tissues compared with adjacent non‐tumor tissues [59]. Although extracellular lactate concentrations in human ESCC tumor interstitial fluid have not yet been directly quantified, Elia et al. directly measured approximately 11 mM lactate in the tumor interstitial fluid of B16‐OVA tumors, compared with approximately 1 mM in plasma [60]. Therefore, the 10 mM NaLa concentration used in our rescue experiments closely approximates lactate levels measured in the tumor interstitial microenvironment and represents a physiologically relevant, tumor‐associated experimental condition. Beyond cell‐intrinsic regulation, lactate is increasingly recognized as a key modulator of the TME, influencing immune suppression and stromal remodeling [60, 61]. Although our study focuses on the epigenetic function of lactate, these findings suggest that metabolic reprogramming may coordinately regulate both tumor cells and the TME, warranting further investigation. Beyond its tumor cell‐intrinsic effects, lactate is increasingly recognized as a critical regulator of the tumor immune microenvironment. Our findings suggest that lactate not only promotes epigenetic reprogramming through the WNK1 signaling axis but may also contribute to the establishment of an immunosuppressive TME characterized by reduced CD8^+^ T‐cell infiltration and enhanced CAF activation. Whether WNK1 signaling directly participates in this process, or whether these effects are primarily mediated by lactate‐dependent metabolic remodeling, remains to be determined. Further studies are therefore needed to clarify the interplay between lactate metabolism, WNK1 signaling, and tumor immune regulation. The mechanism underlying WNK1 nuclear translocation also warrants further investigation. Our findings suggest that lactate promotes the nuclear accumulation of WNK1, potentially through an Importin α5‐dependent nuclear import pathway. However, whether WNK1 contains a functional nuclear localization signal or requires additional transport regulators remains to be determined. Our results further suggest that p300/CBP activity contributes to H3K23la deposition, as pharmacological inhibition of p300/CBP reduced H3K23la levels, WNK1 expression, and p‐H3S10 activation. However, whether additional lactyltransferases participate in this process remains to be determined.

Importantly, our study also provides translational insight. We developed CD03017, a selective WNK1 inhibitor, and demonstrated that pharmacological targeting of WNK1 effectively disrupts the lactate–H3K23la–WNK1–p‐H3S10–AKT axis and suppresses tumor growth in vitro and in PDX models. These findings position WNK1 as a targetable vulnerability and support the therapeutic potential of targeting metabolic‐epigenetic signaling pathways, in line with emerging epigenetic and metabolic therapies.

Despite these findings, several limitations of this study should be acknowledged. First, although we identified a lactate–H3K23la–WNK1–p‐H3S10–AKT axis in ESCC, whether this mechanism is relevant to other cancer types remains to be determined. Moreover, because systemic administration of CD03017 may also inhibit WNK1 in immune cells, future studies will investigate whether WNK1 blockade reshapes antitumor immunity, particularly given the established role of WNK1 in T‐cell adhesion and migration [62]. Finally, although our work focuses on tumor cell‐intrinsic mechanisms, the impact of lactate‐driven epigenetic regulation on the tumor microenvironment warrants further investigation.

In conclusion, our study defines a metabolic‐epigenetic‐signaling axis in ESCC and establishes WNK1 as a key effector linking histone lactylation to oncogenic transcription. This work provides both mechanistic insight and a rationale for therapeutic intervention targeting this pathway.

## 4 Materials and Methods

### 4.1 Cell Lines and Cell Culture Conditions

ESCC cell lines (KYSE30, KYSE450, KYSE150, KYSE510, KYSE70 and KYSE410) were obtained from the Cell Bank of the Chinese Academy of Sciences (Shanghai, China). The immortalized normal esophageal epithelial cell line SHEE was generously provided by Dr. Enmin Li (Shantou University, Guangdong, China) [63, 64]. The ESCC lines were cultured in RPMI‐1640 medium containing 2.0 g/L D‐glucose (11.1 mM), L‐glutamine, and no sodium pyruvate. The Lenti‐293 and HEK‐293 cell lines (ATCC, USA) were cultured in DMEM medium containing 4.5 g/L D‐glucose (25 mM) and 4 mM L‐glutamine without sodium pyruvate. These media were used as the standard culture conditions for the corresponding cell lines, and all control and treatment groups within each cell line were maintained in the same basal medium throughout the experiments. Thus, glucose concentration was kept constant within each experimental comparison, minimizing potential confounding effects of medium composition on the observed treatment‐dependent metabolic changes. All media were supplemented with 10% heat‐inactivated fetal bovine serum (BI) and 1% penicillin/streptomycin (Solarbio). Cells were maintained at 37 °C in a humidified 5% CO_2_ incubator. Authentication of cell lines by cytogenetic analysis was performed every 6 months to ensure cell line identity.

### 4.2 Antibodies and Reagents

Antibody and reagent information is provided in Table S3.

### 4.3 Lactate Level Content Detection

Plasma samples were collected from age‐ and sex‐matched patients with esophageal cancer and healthy individuals in the morning after overnight fasting. All participants were instructed to avoid vigorous exercise before blood collection, and individuals with acute infections, metabolic disorders, or other conditions that could affect lactate metabolism were excluded. Detailed clinical information of the enrolled participants is provided in Table S4. Serum samples and cell culture supernatants were centrifuged at 10 000 × g for 15 min to remove cellular debris. Lactate levels were measured using a lactate assay kit (BC2235, Solarbio) according to the manufacturer's instructions. Absorbance at 570 nm was measured using a spectrophotometer, and lactate concentrations were calculated from the standard curve and normalized to the sample volume. For cell experiments, at the end of each treatment, culture supernatants were collected for lactate quantification, and viable cell numbers were determined in parallel using cell‐counting method. Lactate levels were normalized to the corresponding viable cell number. To minimize potential changes in the pH and osmolarity of the culture medium, cells were treated with NaLa (10 mM) for 24 h.

### 4.4 Western Blot (WB) analysis

Cells and tissues were lysed by incubation with RIPA buffer on ice for 2 h. Protein concentrations were determined by BCA kit (Solarbio, Beijing, China). WB was performed as previously described [65, 66]. Protein bands were visualized by chemiluminescence (ECL) detection reagent (GE Healthcare Life Science, Little Chalfont, HP, UK).

### 4.5 Immunohistochemistry (IHC) Assay and Analysis

IHC staining was performed on a commercial tissue microarray (Shanghai Xinchao Biotechnology Corp.) and PDX tumors. Tissue microarray 1 (HEsoS180Su05) contained 88 tumor tissues and 52 adjacent non‐tumor tissues. Tissue microarray 2 (HEsoS180Su05) contained 96 tumor specimens and 49 adjacent non‐tumor tissues. Tissue microarray 3 (HEsoS180Su05) contained 105 tumor specimens and 50 adjacent non‐tumor tissues. Corresponding clinicopathological information was provided by the manufacturer. Hematoxylin and eosin (H&E) staining was also performed for PDX tumors. IHC staining was performed as previously described [65, 66]. We quantitatively scored the tissue sections based on the percentage of positive cells and staining intensity [67]. For tissue microarrays 1 and 3, protein expression was evaluated according to the percentage of positively stained nuclei in tumor cells. Samples with nuclear staining in ≥ 50% of tumor cells were classified as having high expression, whereas those with < 50% positive nuclei were classified as having low expression. For tissue microarray 2, staining intensity was quantitatively analyzed using ImageJ software (National Institutes of Health, Bethesda, MD, USA). The optical density (OD) of positive staining was measured, and the median OD value of all samples was used as the cutoff to classify tumors into high‐ and low‐expression groups. All stained sections were independently scored by two experienced pathologists who were blinded to the clinicopathological information. In cases of disagreement, the slides were jointly re‐examined to reach a consensus, and the consensus score was used as the final IHC score for statistical analysis.

### 4.6 MTT Assay

Cells were seeded in 96‐well plates at a density of 2000 cells per well in 200 µL of growth medium. Cell proliferation was assessed using the MTT assay. The absorbance at 570 nm was measured using a microplate reader. Three replicates were performed for each cell line at each time point and growth curves were plotted.

### 4.7 Foci Formation Assay

Cells were seeded in 6‐well plates at a density of 600 cells per well in 3 mL of growth medium and incubated at 37 °C for 10 days. After 10 days, colonies were fixed and stained by 0.4% crystal violet solution. Excess stain was rinsed off with PBS and the culture plates were allowed to air dry. Representative images of the stained colonies were captured and the number of colonies was counted manually. The assay was performed in triplicate for each cell line.

### 4.8 Anchorage‐Independent Cell Growth Assay

Cells were suspended in 0.33% agar solution diluted in BME/10% FBS medium at a density of 8000 cells/mL. 1 mL of cell‐agar suspension was layered on top of a 3 mL underlayer of 0.66% agar in each well of a 6‐well plate. Plates were then incubated at 37 °C for 10 days. Subsequently, colonies were visualized under a wide‐field microscope and photographed. The number of colonies larger than 50 µm were counted using Image‐Pro Plus software. The assay was performed in triplicate wells for each cell line.

### 4.9 CUT‐Tag Assay and RNA‐Seq Analysis

According to the manufacturer's instructions, CUT‐Tag detection was performed using the Illumina Pro's Hyperactive Universal CUT‐Tag Detection Kit (TD903, Vazyme Biotech). In brief, approximately 50 000–100 000 fresh cells were incubated with 10 µL of biotin‐coated beads in low‐binding PCR tubes. The antibody buffer containing 1 µg of the target antibody was added, and the mixture was incubated overnight at 4 °C. The microbeads were then washed with Dig‐Wash buffer and incubated with the secondary antibody at room temperature for 60 min. After re‐washing, pA/G‐Tnp was added and rotated for 1 h. A mixture of markers, including a certain amount of spiked control DNA, was then added and incubated at 37 °C for 60 min. DNA was extracted and purified using protease K and DNA Extract Beads. The beads were stored at 55 °C for 30 min and then at ‐20 °C. For library preparation, 50 µL of purified DNA was amplified using the recommended procedure. The amplified library was purified with VAHTS DNA Clean Beads and sequenced on the Illumina NovaSeq platform to generate 150 bp end‐paired reads.

Bioinformatics parameters and details: Signal distributions around transcription start sites (TSSs) and gene bodies were visualized using deepTools (v3.5.1), while genomic annotation of peaks was performed using ChIPseeker. Representative enriched genomic regions were visualized with Gviz. Peak‐associated genes were defined as the genes whose TSSs were closest to the peak centers. Gene Ontology (GO) and Kyoto Encyclopedia of Genes and Genomes (KEGG) enrichment analyses were subsequently performed using clusterProfiler. Motif discovery and enrichment analyses were conducted with the MEME Suite (v5.4.1) against the JASPAR database (http://jaspar.genereg.net/). An R script was used to generate CUT‐Tag signal profiles across gene bodies and their upstream/downstream 3‐kb flanking regions. Consensus peaks were generated by merging peaks detected in biological replicates within each group. Differential peak analysis between groups was performed using DiffBind, with regions meeting the criteria of |log2 fold change| > 0.58 and FDR < 0.05 considered significantly differential peaks (DPs). Hierarchical clustering was then performed based on DP signal intensities across all samples. DP‐associated genes were identified according to the nearest TSS using ChIPseeker, followed by GO and KEGG enrichment analyses using clusterProfiler. Finally, motif enrichment analysis of the differential peaks was performed using the MEME Suite (v5.4.1) against the JASPAR database.

### 4.10 Chromatin Immunoprecipitation (ChIP) Assay

The Simple ChIP Plus Sonication Chromatin IP Kit (CST, #56383) was used for the ChIP assay. The ChIP assay was performed according to the manufacturer's instructions. Specifically, chromatin was fragmented to an average size of approximately 500 bp, with a predominant fragment size centered around 250 bp, and fragmentation efficiency was verified by agarose gel electrophoresis before immunoprecipitation. Sonication was performed using an Ultrasonic Cell Crusher (Nanjing Xinchen Biotechnology Co., Ltd.) at high‐power mode (30% amplitude, 160 W). To minimize sample overheating, chromatin was sonicated for 25 cycles consisting of 30 s ON and 30 s OFF, corresponding to a total active sonication time of 12.5 min. To facilitate the crosslinking of proteins with DNA, 1% formaldehyde was used to treat cells for 10 min at room temperature. The cross‐links were then quenched using a glycine solution. Following this, nuclear lysates were added to digest chromatin and make DNA fragments through sonication. Immunoprecipitation of the nuclear lysate was performed using target antibodies, as well as IgG antibodies as a control. Primers were purchased and qRT‐PCR assays were used to analyze the purified DNA fragments.

### 4.11 Double Luciferase Experiment

The *WNK1* promoter sequence relative to the transcription start site was amplified by PCR from human PBMCs and inserted into the pGL3‐basic vector (PGL3‐WNK1). KYSE450 cells were co‐transfected with pRL‐TK (Promega) and either PGL3‐WNK1 or pGL3‐basic vector, and incubated with or without lactate for luciferase activity analysis. The pGL3 vector containing *WNK1* promoter sequence (pGL3‐WNK1) was constructed as described previously. KYSE30 and KYSE450 cells were co‐transfected with WNK1‐overexpressing (Flag‐WNK1) pcDNA3.1, pGL3‐histone H3, pRL‐TK (Promega) and treated cells with 2‐Deoxy‐D‐glucose (2‐DG) (1 mM) and NaLa (10 mM) for 2 days. Luciferase activity was determined using the DLRTM Assay System (Promega) with a BioTek Synergy H1 microplate reader and the firefly/Renilla ratio was quantified.

### 4.12 WNK1 Conditional Knockout (*Wnk1*‐cKO) Mice Induced Esophageal Cancer Model

*Wnk1*‐cKO mice were purchased from Cyagen Biosciences Inc. *Wnk1*‐cKO mice were generated using the Cre‐LoxP recombination system, which is widely applied for tissue‐specific gene knockout, gene inversion, insertion, and chromosomal translocation. In this study, *Wnk1* ^flox/flox^ mice were crossed with ERT2‐Cre transgenic mice to obtain *Wnk1*‐cKO mice. ERT2‐Cre is an inducible Cre recombinase line in which Cre activity is restricted to epithelial cells and is not active in immune cells, thereby excluding potential confounding effects caused by immune cell‐intrinsic deletion of Wnk1.

Breeding of *Wnk1* ^flox/flox^ mice and ERT2‐Cre mice was performed according to the protocol provided by the manufacturer. Mouse genotypes were identified by PCR analysis. Both *Wnk1*‐cKO mice and wild‐type (WT) littermates were induced to develop esophageal cancer using 4‐Nitroquinoline N‐oxide (4NQO). Briefly, 4NQO was dissolved in drinking water at a concentration of 100 mg/L and administered to mice for 16 weeks, with the solution replaced weekly. After 4NQO treatment, mice were provided with regular drinking water for an additional 12 weeks. Control mice were continuously supplied with normal drinking water for 28 weeks. At the end of the experiment, mice were sacrificed, and esophageal tissues were harvested for subsequent analyses.

The following primers were used for detecting mouse genotypes by PCR assay, the primer sequences are listed in Table S5.

### 4.13 Knockdown Assay

Lentivirus particles containing control (pLKO.1) or target gene short hairpin RNA (shRNA) were generated by co‐transfecting lenti‐293 cells with shRNA plasmid, psPAX2 and pMD2.G packaging plasmids using Lipofectamine 2000 according to the manufacturer's instructions. ESCC cell lines were infected with lentivirus in the presence of 8 µg/mL polybrene. Infected cells were selected with 4 µg/mL puromycin for 24 h. Positively selected cells were used for subsequent experiments. The shRNA target sequences were provided in Table S6.

### 4.14 Co‐Immunoprecipitation (Co‐IP) Experiment

HEK‐293T cells were transiently transfected with plasmids (5 µg) for 48 h. Cell lysates (600 µg) were immunoprecipitated with anti‐tag antibodies (1 µg) and resolved using WB to detect the binding between proteins. Additionally, endogenous Co‐IP experiments were conducted in ESCC cell lines. Cell lysates (600 µg) were immunoprecipitated with anti‐target antibody (1 µg) and normal IgG antibody (1 µg) to assess the binding between proteins via WB experiment.

### 4.15 Immunofluorescence (IF) Assay

Cells were seeded onto glass coverslips in 24‐well plates. After overnight adhesion, cells were fixed with 4% paraformaldehyde and permeabilized with 0.3% Triton X‐100. Cells were then incubated overnight at 4 °C with primary rabbit anti‐WNK1 (1:100) and mouse anti‐histone H3 (1:100) antibodies diluted in PBS containing 3% BSA and 10% goat serum. The next day, cells were washed with PBS and incubated with Alexa Fluor 594‐conjugated goat anti‐mouse IgG (red fluorescence) and Alexa Fluor 488‐conjugated goat anti‐rabbit IgG (green fluorescence) secondary antibodies for 1 h at room temperature. After washing, coverslips were mounted onto glass slides using a fluorescent mounting medium containing DAPI. 3D imaging was performed using a Nikon A1R confocal laser scanning microscope. WNK1 and histone H3 fluorescence signals were detected in separate channels; pixel overlay of the two channels was used to determine protein co‐localization. Nuclear staining was included to visualize cell morphology.

### 4.16 EdU Cell Proliferation Assay

Cell proliferation was assessed using an EdU Cell Proliferation Kit with Alexa Fluor 488 (Beyotime, C0071S). KYSE150 and KYSE450 cells were seeded in 24‐well plates and treated as indicated. Cells were incubated with 10 µM EdU for 2 h at 37 °C, fixed with 4% paraformaldehyde for 15 min, permeabilized with 0.3% Triton X‐100 for 15 min, and subjected to the Click reaction according to the manufacturer's instructions. Nuclei were counterstained with DAPI, and images were captured using a fluorescence microscope. The percentage of EdU‐positive cells was calculated as the number of EdU‐positive cells divided by the total number of DAPI‐positive cells. All experiments were performed independently three times.

### 4.17 Wound‐Healing Assay

KYSE30 and KYSE450 cells transduced with shNC or shWNK1 #1 were seeded in 6‐well plates and cultured to form a confluent monolayer. A linear scratch was generated using a sterile 200 µL pipette tip. Detached cells were removed by gentle washing with PBS, and the cells were subsequently cultured in low‐serum medium. Images of the same fields were captured at 0, 24, and 48 h. The wound area was quantified using ImageJ, and the wound‐closure rate was calculated as follows: (wound area at 0 h – wound area at the indicated time point)/wound area at 0 h × 100%. All experiments were independently repeated three times.

### 4.18 Transwell Migration Assay

KYSE30 and KYSE450 cells transduced with shNC or shWNK1 #1 were suspended in serum‐free medium and seeded into the upper chambers of Transwell inserts, while complete medium containing serum was added to the lower chambers as a chemoattractant. After incubation, non‐migrated cells on the upper surface were removed, and cells that migrated to the lower surface of the membrane were fixed and stained with crystal violet. Migrated cells were imaged and counted in randomly selected fields. Relative migration rates were normalized to the shNC group. All experiments were performed independently three times.

### 4.19 Transwell Invasion Assay

KYSE30 and KYSE450 cells transduced with shNC or shWNK1 #1 were seeded into the upper chambers of Transwell inserts precoated with Matrigel, and complete medium containing serum was added to the lower chambers. After incubation, non‐invading cells were removed from the upper surface, whereas cells that invaded through the Matrigel and reached the lower surface of the membrane were fixed and stained with crystal violet. Invaded cells were imaged and counted in randomly selected fields. Relative invasion rates were normalized to the shNC group. All experiments were performed independently three times.

### 4.20 ChIP‐re‐ChIP Assay

ChIP‐re‐ChIP assays were performed to evaluate the co‐occupancy of p‐H3S10 and H3K23la at the *WNK1* promoter. Cross‐linked chromatin was sonicated and first immunoprecipitated with an anti‐p‐H3S10 antibody overnight at 4 °C. After capture with protein A/G beads and extensive washing, the immunoprecipitated chromatin was eluted and subjected to a second immunoprecipitation with an anti‐H3K23la antibody. The resulting DNA was purified after reversal of cross‐links and analyzed by qPCR using primers specific for the *WNK1* promoter. Enrichment was normalized to input DNA and IgG controls. All experiments were independently repeated three times.

### 4.21 Nuclear and Cytoplasmic Protein Extraction Assay

Nuclear and cytoplasmic proteins were isolated using a Nuclear and Cytoplasmic Protein Extraction Kit (Beyotime, P0028) according to the manufacturer's instructions. KYSE30 and KYSE450 cells were treated with DMSO, 2‐DG (1 mM), or NaLa (10 mM) for 24 h, harvested, and subjected to sequential extraction of cytoplasmic and nuclear proteins. Protein concentrations were determined using a BCA protein assay, and equal amounts of protein were analyzed by Western blotting. β‐actin and Lamin B1 were used as cytoplasmic and nuclear marker proteins, respectively, to verify the purity of the subcellular fractions.

### 4.22 Cell Cycle Analysis

Cell cycle distribution was analyzed by flow cytometry. Cells were fixed in prechilled 70% ethanol at 4 °C overnight. After washing with PBS, the cells were permeabilized with 0.3% Triton X‐100 for 60 min and then incubated with an anti‐phospho‐histone H3 (Ser10) (p‐H3S10) antibody, an M‐phase marker, at 4 °C overnight. After washing three times with PBS, the cells were incubated with an Alexa Fluor 647‐conjugated secondary antibody (Invitrogen, A21245) for 1 h at room temperature. The cells were then washed three times with PBS and incubated with RNase A and propidium iodide (PI) staining solution for 30 min at room temperature in the dark. DNA content and p‐H3S10 staining were analyzed by flow cytometry, and the percentages of cells in the G1, S, G2, and M phases were determined using the corresponding flow cytometry analysis software. All experiments were independently performed three times.

### 4.23 Apoptosis Assay

Cell apoptosis was assessed by flow cytometry using Annexin V‐FITC/propidium iodide (PI) staining. Briefly, cells transduced with shNC or shWNK1 #1 were harvested, washed with PBS, and resuspended in binding buffer. The cells were then stained with Annexin V‐FITC (BioLegend, San Diego, CA, USA) and PI according to the manufacturer's instructions. After incubation in the dark, the stained cells were analyzed using a FACSCalibur flow cytometer (BD Biosciences, San Jose, CA, USA). Annexin V‐positive/PI‐negative cells were considered early apoptotic cells, whereas Annexin V‐positive/PI‐positive cells were considered late apoptotic cells. The total apoptotic rate was calculated as the percentage of early and late apoptotic cells. All experiments were independently performed three times.

### 4.24 Single‐Cell RNA‐Sequencing Data Analysis

The ESCC scRNA‐seq dataset GSE160269 was downloaded from the Gene Expression Omnibus (GEO) database and analyzed using the Seurat package (v4.4.0) in R (v4.4.1). Cells with fewer than 2,000 detected genes were excluded from further analysis. The remaining data were normalized using the LogNormalize method, and the top 2,000 highly variable genes were identified using the FindVariableFeatures function. The normalized expression matrix was scaled, followed by principal component analysis (PCA). The first 50 principal components were used to construct a shared nearest‐neighbor graph and perform unsupervised clustering using the FindNeighbors and FindClusters functions, with the clustering resolution set to 1.0. Uniform Manifold Approximation and Projection (UMAP) was subsequently performed for dimensionality reduction and visualization. Cell types were annotated based on canonical marker genes and the published annotations of the GSE160269 dataset. The expression patterns of LDHA, WNK1, and AKT1 were evaluated across different cell populations. Samples were stratified into LDHA‐high and LDHA‐low groups according to the median LDHA expression level, and the proportions of major immune and stromal cell populations were compared between the two groups. Statistical comparisons were performed using the Wilcoxon rank‐sum test, and a two‐sided P < 0.05 was considered statistically significant.

### 4.25 Multiplex Immunofluorescence Staining

Multiplex immunofluorescence staining was performed on formalin‐fixed, paraffin‐embedded tumor sections using a four‐color multiplex fluorescent immunohistochemistry kit (CSF1003, 50 tests; Celnovte Biotechnology, Suzhou, China) according to the manufacturer's instructions. Following deparaffinization, antigen retrieval, and endogenous peroxidase blocking, tissue sections were sequentially incubated with primary antibodies, HRP‐conjugated secondary antibodies, and TSA fluorophores. Heat‐mediated stripping was performed after each staining cycle to enable sequential labeling of multiple targets. After nuclear counterstaining with DAPI, images were acquired using a multispectral fluorescence microscope under identical imaging settings for all samples.

### 4.26 In Vitro Kinase Assay

Kinase assay was performed according to the manufacturer's instructions. Human recombinant histone H3 protein was used as the substrate for WNK1, Histone H3 was mixed with active WNK1 for the in vitro kinase assay. 200 µM final concentration of ATP and kinase buffer (CST, #9802) were added to mixtures, and incubated them at 30 °C for 50 min. Then reactions were stopped by the addition of 5×loading buffer and results were analyzed by WB experiment.

### 4.27 Oxygen Consumption and ECAR Experiment

Extracellular acidification rates (ECAR) of ESCC cells were analyzed using a Seahorse XF24 Extracellular Flux Analyzer (Agilent). Cells were seeded at 30,000 cells/well on a 24‐well Seahorse XF24 plate and allowed to adhere overnight. Prior to analysis, growth medium was replaced with assay medium. Using pre‐loaded cartridges, glucose (10 mM), oligomycin (1 µM), and 2‐DG (50 mM) were sequentially injected into wells at specified timepoints. ECAR measurements were recorded throughout and data were analyzed using Seahorse XF24 software. ECAR measures lactic acid secretion reflecting glycolytic flux.

### 4.28 Cellular Thermal Shift Assay (CETSA)

Prepare HEK293 cells and transfect them with the WNK1 expression plasmid using Lipofectamine 2000 reagent. Continue culturing the cells for 48 h. After 48 h, treat the cells with CD03017 which was purchased from AnyMol (Taosu Biotechnology, China. https://www.anymole.com/hitinfo/HIT212624717) for 4 h, then collect the cells and aliquot them into PCR tubes (100 µL/tube). Incubate the samples in a thermal gradient ranging from 40–56 °C, with 2 °C increments for 5 min. After performing two freeze‐thaw cycles in liquid nitrogen, collect the cell supernatants and analyze WNK1 expression by WB.

### 4.29 Cell‐Derived Xenograft (CDX) and Patient‐Derived Xenograft (PDX)Mouse Model

All animal studies were approved by the Ethics Committee of the China‐US (Henan) Hormel Cancer Institute (No. CUHCI2019070). All patient samples were approved by the Ethics Committee of the Affiliated Cancer Hospital of Zhengzhou University (Zhengzhou, Henan, China; No. 2023‐KY‐0156‐001). Cell line‐derived and patient‐derived xenograft mouse models were used to evaluate target gene knockdown on esophageal tumor growth in vivo.

For CDX model, target gene knockdown, control knockdown, and parental esophageal cancer cells were generated as described previously [68]. 8–10‐week‐old female athymic nude mice were subcutaneously injected with 1×10^7^ cells of each cell line. Tumor volumes were measured weekly by caliper and calculated as 0.5×length×width×height.

For PDX model, primary human esophageal tumor tissues were serially passaged in NOD‐SCID mice. When tissues reached 4–6 passages, 0.1 g tumor fragments were cut and subcutaneously implanted in mice. When xenografts reached approximately 150 mm^3^, mice were treated with PBS or CD03017 (25 mg/kg & 100 mg/kg) via intraperitoneal injection for approximately 3 weeks; mice were intratumorally treated twice weekly over a duration of 3 weeks with lentivirus encoding shWNK1 or control shRNA. Tumor growth was monitored weekly by caliper measurements. Mice were euthanized when a significant difference in tumor size was observed and tumors were harvested for further analysis; the tumor harvest time point was determined according to recommended guideline [69]. All animal procedures followed ethical regulations and guidelines for humane care.

### 4.30 Compound Screening

Molecular docking screening used Autodock Vina software to place 53,344 ligands from the Maybridge compound library into WNK1 pocket. Ligand conformations were searched and adjusted to identify potential binding conformations. Each binding conformation was scored and evaluated, and the best‐scoring conformation was selected. After docking scoring, the docking binding energy (affinity) was analyzed. Negative affinity values indicate a strong intermolecular interaction and are generally used to measure the tightness of ligand‐receptor binding; larger negative values indicate stronger binding. Here, an affinity > −4 kcal/mol indicates very weak or no binding; −7 kcal/mol < Affinity ≤ −4 kcal/mol indicates moderate binding; and Affinity ≤ −7 kcal/mol indicates strong binding. Compounds with an affinity ≤ −7 kcal/mol were selected for subsequent validation.

### 4.31 LC‐MS/MS Analysis

Quantitative analysis was conducted using an Agilent 1260 HPLC system coupled to a 6460C triple quadrupole mass spectrometer (Agilent). Separation was achieved on a 100 mm × 3.0 mm, 3.5 µm analytical column. The column temperature was maintained at 35 °C. The mobile phase consisted of water (A) and acetonitrile (B), with isocratic elution at 90% acetonitrile for 8 min at a flow rate of 0.3 mL/min. Mass spectrometric detection was performed in negative ion mode using multiple reaction monitoring (MRM). Plasma Sample Preparation: After intraperitoneal administration in mice, plasma samples were collected at predetermined time points. 40 µL aliquot of plasma was mixed with 200 µL of anhydrous acetonitrile, vortexed thoroughly, and incubated on ice for 20 min. The mixture was centrifuged at 12 000 rpm for 10 min at 4 °C, and the supernatant was collected. The extract was evaporated to dryness under vacuum and reconstituted in 80% acetonitrile prior to LC‐MS/MS analysis.

### 4.32 Open Field Test

Mice were administered CD03017 via intraperitoneal injection (0, 25, 100 mg/kg). Behavioral experiments were conducted 7 days after administration, following the methods described in reference [70].

### 4.33 Gene Set Enrichment Analysis (GSEA)

GSEA was performed to assess pathway‐level metabolic alterations across multiple cancer types. Gene expression data (FPKM values) for tumor tissues and matched adjacent normal tissues were obtained from the Gene Expression Omnibus (GEO) database using publicly available datasets: esophageal squamous cell carcinoma (ESCC; GSE180791), stomach adenocarcinoma (STAD; GSE27342), and liver hepatocellular carcinoma (LIHC; GSE154964). Detailed sample information, including the number of tumor and normal samples in each dataset.

Raw expression matrices were processed and normalized using R. Data preprocessing, normalization, and visualization were performed using the dplyr and ggplot2 packages. Genes were ranked based on differential expression between tumor and normal samples for each cancer type.

GSEA was conducted using the GSEA software (Broad Institute with KEGG pathway gene sets as the reference database. A total of 1,000 permutations were performed using the phenotype permutation method. Enrichment significance was evaluated using the normalized enrichment score (NES) and false discovery rate (FDR). Pathways with an FDR < 0.25 and a nominal P value < 0.05 were considered significantly enriched.

### 4.34 Statistical Analysis

Quantitative data are expressed as mean ± SD from at least three independent experiments. Statistical analyses were performed using the SPSS software. Differences between experimental groups were analyzed by One‐way ANOVA followed by appropriate post‐hoc tests. *p* < 0.05 was considered statistically significant.

## Author Contributions

M.Y.Y, S.B.F and Y.M.S conceived and designed the experiments; M.Y.Y, S.B.F and G.G.J assisted with designing the figures and writing; L.Z, K.X.Z, Z.W.W, M.L and Y.S helped to inoculate the PDX mice and X.Y.J and Q.X.G helped to breed knockout mice; M.H.L, G.G.J and Z.G.D edited the manuscript, supervised the studies and allocated the funding.

## Funding

This study was supported by the National Natural Science Foundation of China (No. 82573804), the Natural Science Foundation of Henan Province (No. 262300421618), and grants from the Henan Cardiovascular Disease Center (Central China Subcenter of the National Center for Cardiovascular Diseases) (No. 2023‐FZX14), Medical Science and Technology Project of Henan Province, China (SBGJ202602022), as well as the Henan Province International Science and Technology Cooperation Program (No. 262102520087 and 262102520088).

## Ethics Statement

All animal experiments and clinical samples in this project were conducted in accordance with the Declaration of Helsinki and were approved by the Ethics Committee of the China‐US (Henan) Hormel Cancer Institute (Zhengzhou, Henan, China). All cancer tissues used in the study were obtained from cancer patients.

## Conflicts of Interest

The authors declare no conflicts of interest.

## Supporting information




**Supporting File 1**: advs78438‐sup‐0001‐SuppMat.docx.

**Supporting File 2**: advs78438‐sup‐0002‐TableS1.docx.

**Supporting File 3**: advs78438‐sup‐0003‐TableS2.docx.

**Supporting File 4**: advs78438‐sup‐0004‐TableS3.docx.

**Supporting File 5**: advs78438‐sup‐0005‐TableS4.docx.

**Supporting File 6**: advs78438‐sup‐0006‐TableS5.docx.

**Supporting File 7**: advs78438‐sup‐0007‐TableS6.docx.

## Data Availability

The data that support the findings of this study are available on request from the corresponding author. The data are not publicly available due to privacy or ethical restrictions.
