# Supplementary material for: Histone Lactylation Drives WNK1 Expression to Promote Esophageal Squamous Cell Carcinoma Progression Through Histone H3 Ser10 Phosphorylation
Source: Adv Sci (Weinh). 2026 Oct 25:e78438. Online ahead of print. doi: 10.1002/advs.78438 (PMC13696526; doi:10.1002/advs.78438)
Supplement: Supplementary file 1 [file ADVS-9999-e78438-s001.docx]

Supplementary Table 3. Antibody and reagent

| Antibody and Reagent | | | Source | Identifier | Application |
| --- | --- | --- | --- | --- | --- |
| Pan-Klac | | | PTMBIO | **PTM-1401RM** | **WB:1:1000** |
| H3K9lac | | | PTMBIO | **PTM-1419RM** | **WB:1:1000** |
| H3K14lac | | | PTMBIO | **PTM-1414RM** | **WB:1:1000** |
| H3K18lac | | | PTMBIO | **PTM-1406RM** | **WB:1:1000** |
| H3K23lac | | | PTMBIO | **PTM-1413RM** | **WB:1:1000;IHC:1:800** |
| H3K27lac | | | PTMBIO | **PTM-1428** | **WB:1:1000** |
| H3K56lac | | | PTMBIO | **PTM-1421RM** | **WB:1:1000** |
| p-histone H3 (Thr12) | | | Affinity | **#AF7336** | **WB:1:1000** |
| p-histone H3 (Ser28) | | | Affinity | **#AF3868** | **WB:1:1000** |
| p-histone H3 (Ser58) | | | Affinity | **#AF7335** | **WB:1:1000** |
| Ki67 | | | HUABIO | **HA721115** | **IHC:1:1000** |
| Anti-flag | | | HUABIO | **M1403-2** | **WB:1:1000** |
| Pan-CK | | | HUABIO | **ER1914-74** | **IF:1:100** |
| p-AKT (S473) | Cell Signaling Technology | | | **#4060** | **WB:1:1000;IHC:1:100** |
| AKT | Cell Signaling Technology | | | **#9272** | **WB:1:1000** |
| GAPDH | Cell Signaling Technology | | | **#97166** | **WB:1:3000** |
| Histone H3 | Cell Signaling Technology | | | #9715 | **WB:1:1000** |
| p-histone H3 (Ser10) | Cell Signaling Technology | | | #9701 | **WB:1:1000** |
| p-histone H3 (Thr3) | Cell Signaling Technology | | | #9714 | **WB:1:1000** |
| Histone H3 | Cell Signaling Technology | | | #14269S | **IF:1:100** |
| p-histone H3 (Ser10) | Cell Signaling Technology | | | #53348 | **CHIP:1:50** |
| Normal rabbit IgG | Cell Signaling Technology | | | #2729 | IP: 1:200; CHIP:1:50 |
| EGFP | Abcam | | | ab184601 | **IP:1:200** |
| WNK1 | Abcam | | | **ab137687** | **WB:1:1000;IHC:1:100** |
| p-histone H3 (Ser10) | Abcam | | | ab267372 | **WB:1:1000** |
| beta-actin-HRP | Proteintech | | | HRP-60008 | **WB:1:1000** |
| WNK1 | Proteintech | | | 28357-1-AP | **IF:1:100** |
| Importin alpha5 | Proteintech | | | 10077-1-AP | **WB:1:1000** |
| Importin beta1 | Proteintech | | | 18137-1-AP | **WB:1:1000** |
| CD8 alpha | Proteintech | | | 66868-1-Ig | **IF:1:100** |
| FAP | Proteintech | | | 11779-1-AP | **IF:1:100** |
| Flag-tag | Sigma-Aldrich | | | F1804 | **WB:1:1000** |
| HRP-goat anti-mouse | Beijing Zhongshan Jinqiao Biotechnology Corp | | | ZB-2305 | **WB:1:3000** |
| HRP-goat anti-Rabbit | Beijing Zhongshan Jinqiao Biotechnology Corp | | | ZB-5301 | **WB:1:3000** |
| Alexa Fluor 594-conjugated goat anti- mouse IgG | Thermo Fisher Scientific | | | A11005 | **IF:1:100** |
| Alexa Fluor 488-conjugated goat anti-rabbit IgG | | Thermo Fisher Scientific | | A11008 | **IF:1:100** |
| Lipofectamine 2000 | | Thermo Fisher Scientific | | 11668019 |  |
| TRIzol™ reagent | | Thermo Fisher Scientific | | 15596026CN |  |
| Epoxy-activated Sepharose 6B beads | | Cytiva | | 17048001 |  |
| CM5 sensor chip | | Cytiva | | BR100530 |  |
| Esophageal Cancer Organoid Kit | | bioGenous | | K2177-ES |  |
| L-Lactate level kit | | Solarbio | | BC2235 |  |
| Multi-IF Kit | | Celnovte | | CSF1003 |  |
| Nuclear and Cytoplasmic Protein Extraction Kit | | Beyotime | | P0028 |  |
| EdU cell proliferation Kit with AF488 | | Beyotime | | C0071S |  |
