# Supplementary material for: Histone Lactylation Drives WNK1 Expression to Promote Esophageal Squamous Cell Carcinoma Progression Through Histone H3 Ser10 Phosphorylation
Source: Adv Sci (Weinh). 2026 Oct 25:e78438. Online ahead of print. doi: 10.1002/advs.78438 (PMC13696526; doi:10.1002/advs.78438)
Supplement: Supplementary file 2 [file ADVS-9999-e78438-s002.docx]

| Supplementary Table 5. Primers were used for detecting mouse genotypes | | |
| --- | --- | --- |
| Name | Sequence |  |
| WNK1 |  | |
| Forward primer: | 5'- AAGATACAGTACTCTGTTCTCCAAC -3' | |
| Reverse primer: | 5'- AAAGTCATTTGCGTGCCTTAAGAG -3' | |
| ESCC Cre |  | |
| Forward primer: | 5'-AATGTTAATCCATATTGGCAGAACG -3' | |
| Reverse primer: | 5'-TGCATGATCTCCGGTATTGAAACT-3' | |
