# Supplementary material for: Histone Lactylation Drives WNK1 Expression to Promote Esophageal Squamous Cell Carcinoma Progression Through Histone H3 Ser10 Phosphorylation
Source: Adv Sci (Weinh). 2026 Oct 25:e78438. Online ahead of print. doi: 10.1002/advs.78438 (PMC13696526; doi:10.1002/advs.78438)
Supplement: Supplementary file 3 [file ADVS-9999-e78438-s003.docx]

| Supplementary Table 6. sequence for knockdown | | |
| --- | --- | --- |
| Name | Sequence |  |
| shWNK1 |  | |
| shWNK1 #1: | 5'- GCAGGAGTGTCTAGTTATATT -3' | |
| shWNK1 #2: | 5'- CAGCCTTATGTGGAATCAAAT -3' | |
| shHistone3.3 |  | |
| shHistone3.3A: | 5'- CCATGCCAAACGTGTAACAAT-3' | |
| shHistone3.3B: | 5'- CGCTAAGAGAGTCACCATCAT -3' | |
