# Supplementary material for: Histone Lactylation Drives WNK1 Expression to Promote Esophageal Squamous Cell Carcinoma Progression Through Histone H3 Ser10 Phosphorylation
Source: Adv Sci (Weinh). 2026 Oct 25:e78438. Online ahead of print. doi: 10.1002/advs.78438 (PMC13696526; doi:10.1002/advs.78438)
Supplement: Supplementary file 4 [file ADVS-9999-e78438-s004.docx]

**Supplementary Information**

**Histone lactylation drives WNK1 expression to promote esophageal squamous cell carcinoma progression through histone H3 Ser10 phosphorylation**

**Supplementary Figure 1. Glycolysis inhibition suppresses ESCC cell proliferation in vitro and tumor growth in vivo.**

**A-C.** MTT assays showing the effects of DCA, Oxamate, and 2-DG on the proliferation of KYSE30 and KYSE450 cells. **D**. MTT assays were performed to evaluate the effects of glycolysis inhibitors on the proliferation of SHEE cells. **E-J**. Representative colony formation assays and corresponding quantification showing the effects of DCA, Oxamate, and 2-DG on the clonogenic growth of KYSE30 and KYSE450 cells (n=3). **K.** MTT assays showing that NaLa partially rescued the inhibitory effect of 2-DG on ESCC cell proliferation. **L-M**. Representative colony formation assays and corresponding quantification showing that NaLa partially rescued the inhibitory effect of 2-DG on clonogenic growth (n=3). **N.** MTT assays showing that depletion of endogenous histone H3 markedly inhibited the proliferation of KYSE30 and KYSE450 cells. Re-expression of wild-type H3 or H3 mutants carrying K9A, K14A, or K18A substitutions largely restored cell proliferation, whereas the H3K23A mutant failed to rescue the growth defect (n=3). **O-P.** Representative colony formation assays and corresponding quantification showing that re-expression of wild-type H3, H3K9A, H3K14A, or H3K18A restored clonogenic growth following H3 depletion, whereas H3K23A failed to rescue colony formation, indicating an essential role of H3 Lys23 in ESCC cell proliferation (n=3). **Q-R**. PDX experiments were conducted to assess the effect of NaLa treatment on 2-DG-induced ESCC tumor growth inhibition; tumor weights were recorded and analyzed (n=7). **S-T**. Representative IHC staining and corresponding quantification of Ki67 and H3K23la in xenograft tumors from the indicated treatment groups (n=7). **U-V**. Representative images and quantification of tumor weights from CDX models showing that re-expression of wild-type H3, but not H3K23A, restored tumor growth following endogenous H3 depletion (n=5). Data are presented as mean ± SD from three independent experiments unless otherwise indicated. Statistical significance was determined by one-way ANOVA followed by Tukey's multiple-comparison test. **p* < 0.05; ***p* < 0.01; ****p* < 0.001.

**Supplementary Figure 2.** **Lactate promotes WNK1 transcription by increasing H3K23 lactylation at the *WNK1* promoter.**

**A.** Schematic representation of three regions within the ***WNK1*** promoter (Promoters 1-3). **B-E.** Luciferase reporter assays were performed in KYSE30, KYSE450, KYSE150, and KYSE510 cells treated with vehicle, NaLa, or 2-DG to evaluate the transcriptional activity of different regions of the WNK1 promoter. **F-G.** ChIP-qPCR analysis of H3K23ac enrichment at the *WNK1* promoter in KYSE30 and KYSE450 cells following NaLa or 2-DG treatment. **H-I.** KYSE30 and KYSE450 cells were treated with the MCT1 inhibitor AZD3965 in the presence or absence of NaLa. Luciferase reporter assay analysis (H) and immunoblotting (I) showed that AZD3965 blocked WNK1 upregulation. **J.** Schematic representation of the ***WNK1*** promoter deletion construct, showing the deleted region within the *WNK1* promoter. **K-L.** Luciferase reporter assay analysis of H3K23la enrichment at the indicated regions of *WNK1* promoter deletion constructs in KYSE30 and KYSE450 cells treated with NaLa or 2-DG. **M-N.** Luciferase reporter assay analysis of H3K23la enrichment at the *WNK1* promoter in H3K23A-expressing KYSE30 and KYSE450 cells treated with NaLa or 2-DG. Data are presented as mean ± SD from three independent experiments unless otherwise indicated. Statistical significance was determined using one-way ANOVA followed by Tukey's multiple-comparison test. ns, no significant difference.

**Supplementary Figure 3. WNK1 promotes malignant phenotypes of ESCC cells in vitro.**

**A.** WB was performed to measure the level of WNK1 in SHEE and ESCC cell lines. **B.** MTT assays showing that WNK1 knockdown inhibited the proliferation of KYSE30 and KYSE450 cells. **C-D.** Representative soft agar colony formation assays and corresponding quantification showing that WNK1 knockdown impaired anchorage-independent growth of KYSE30 and KYSE450 cells. **E-F.** Representative EdU immunofluorescence images and corresponding quantification showing reduced EdU incorporation following WNK1 knockdown in KYSE450 and KYSE150 cells. **G-H.** Representative wound-healing assays and corresponding quantification showing reduced wound closure following WNK1 knockdown in KYSE30 and KYSE450 cells. **I-J.** Representative Transwell migration assays and corresponding quantification showing that WNK1 knockdown suppressed migration of KYSE30 and KYSE450 cells. **K-L.** Representative Matrigel-coated Transwell invasion assays and corresponding quantification showing that WNK1 knockdown suppressed invasion of KYSE30 and KYSE450 cells. **M-N.** Representative flow cytometric analysis and corresponding quantification of cell apoptosis in KYSE30 and KYSE450 cells following WNK1 knockdown. **O.** WB analysis confirming WNK1 knockdown and ectopic WNK1 expression in ESCC cells. **P.** MTT assays showing that ectopic WNK1 expression restored the proliferation of WNK1-depleted ESCC cells. **Q-R.** Representative colony formation assays and corresponding quantification showing that ectopic WNK1 expression restored clonogenic growth following WNK1 knockdown. Data are presented as mean ± SD from three independent experiments unless otherwise indicated. Statistical significance was determined using Student's t-test or one-way ANOVA followed by Tukey's multiple-comparison test, as appropriate. ns, no significant difference, ***p* < 0.01, ****p* < 0.001.

**Supplementary Figure 4. WNK1 depletion and re-expression do not affect cell-cycle distribution in ESCC cells.**

**A-B.** Representative flow cytometry profiles showing cell-cycle distribution and mitotic-cell populations in KYSE150 cells transduced with shNC (A) or shWNK1 (B). DNA content was assessed by propidium iodide (PI) staining to determine the proportions of cells in G1, S, and G2 phases, whereas mitotic cells were identified by p-H3S10 staining detected in the APC channel.

**C.** Quantification of the proportions of KYSE150 cells in G1, G2, S, and M phases following WNK1 knockdown. **D-G.** Representative flow cytometry profiles showing cell-cycle distribution and p-H3S10-positive mitotic-cell populations in KYSE150 cells transduced with shNC plus empty vector (D), shWNK1 plus empty vector (E), shWNK1 plus wild-type WNK1 (WT) (F), or shWNK1 plus WNK1-D368A (G). **H.** Quantification of the proportions of cells in G1, G2, S, and M phases under the indicated conditions. Re-expression of either WNK1-WT or WNK1-D368A in WNK1-depleted cells did not significantly alter cell-cycle distribution. Data are presented as mean ± SD from three independent experiments. Statistical significance was determined using a two-tailed unpaired Student’s t-test (C) and one-way ANOVA followed by Šídák’s multiple-comparisons test (H). ns, no significant difference.

**Supplementary Figure 5. WNK1 selectively phosphorylates histone H3 at Ser10 and promotes ESCC cell proliferation through H3S10 phosphorylation.**

**A.** MTT assays showing that histone H3 knockdown inhibited ESCC cell proliferation, whereas re-expression of H3 WT or the phosphomimetic mutant H3 S10D, but not the non-phosphorylatable mutant H3 S10A, restored proliferative capacity (n=3). **B-C.** Representative colony formation images and quantification demonstrating that H3 S10D, but not H3 S10A, rescued the impaired clonogenic growth caused by histone H3 depletion. **D.** MTT assays showing that WNK1 knockdown suppressed ESCC cell proliferation, whereas expression of H3 S10D, but not H3 WT or H3 S10A, largely restored cell growth. **E-F.** Representative colony formation images and quantification confirming that the proliferative defect induced by WNK1 depletion was rescued by H3 S10D but not H3 S10A. **G.** MTT assays showing that glycolytic inhibition with 2DG suppressed ESCC cell proliferation, whereas H3 S10D, but not H3 WT or H3 S10A, partially reversed the inhibitory effect. **H-I.** Representative colony formation images and quantification showing that expression of H3 S10D restored colony-forming ability following 2DG treatment, whereas H3 S10A failed to do so. **J.** Cytoplasmic and nuclear fractionation followed by WB analysis showing that glycolytic inhibition by 2-DG decreases nuclear accumulation of WNK1, while NaLa treatment increased the nuclear abundance of WNK1 in KYSE30 and KYSE450 cells. Lamin B1 and β-actin were used as nuclear and cytoplasmic loading controls, respectively. **K-L.** Representative IF images and quantification of WNK1 subcellular localization in KYSE450 cells following treatment with control, 2-DG, or NaLa. The nuclear-to-cytoplasmic fluorescence intensity ratio (Fn/c) of WNK1 was quantified. Statistical significance is indicated by the corresponding p values. **M.** Co-IP analysis showing that WNK1 interacted with Importin α5. **N-O.** IF result showing WNK1 subcellular localization in KYSE450 cells under control conditions or following NaLa treatment in the presence of DMSO or ivermectin (IVM). Data are presented as mean ± SD from three independent experiments. Statistical significance was determined by one-way ANOVA. ns, no significant difference, **p* < 0.05, ***p* < 0.01, ****p* < 0.001.

**Supplementary Figure 6.** **Glycolytic regulation of the H3K23la–WNK1–p-H3S10 axis and its association with AKT signaling in ESCC.**

**A.** WB analysis showing that treatment with the glycolytic inhibitors DCA, Oxamate, and 2-DG dose-dependently reduced H3K23la, WNK1, and p-H3S10 levels in KYSE30 and KYSE450 cells. **B.** Patient inclusion and cohort allocation for IHC and survival analyses. **C.** ChIP-qPCR analysis showing that H3K23la enrichment at the *AKT1* promoter was not significantly altered in KYSE30 or KYSE450 cells. **D.** WB experiment was performed to measure the level of p-AKT(S473) and p-mTOR(S2448) following treatment with 2-DG or NaLa. **E.** Correlation analysis showing significant positive association between *WNK1* and *AKT1* mRNA expression in ESCC. **F-G.** Representative IHC staining images and quantification showing that WNK1 knockdown significantly decreased p-H3S10 and p-AKT(S473) expression in ESCC xenograft tumors (n=6). **H-I.** Representative IHC staining images and quantification showing reduced p-H3S10 and p-AKT(S473) expression in esophageal tumors from *Wnk1*-cKO mice compared with WT mice following 4NQO treatment (n=7). **J.** Kaplan–Meier analysis showing that high WNK1 expression was associated with poorer overall survival in ESCC patients. **K.** Kaplan–Meier analysis showing that elevated PI3K–AKT signaling pathway activity was associated with unfavorable overall survival in ESCC patients. **L.** WB analysis showing that pharmacological inhibition of AKT with MK-2206 reduced p-AKT(S473) but did not markedly alter WNK1 or p-H3S10 expression, suggesting that AKT acts downstream of the WNK1–H3S10 signaling axis. **M.** WB analysis of H3K23la, WNK1, p-H3S10, and p-AKT(S473) levels in KYSE30 and KYSE450 cells treated with the p300/CBP inhibitor A485 at the indicated concentrations. Data are presented as mean ± SD from three independent experiments. Statistical significance was determined using one-way ANOVA. ns, no significant difference.

**Supplementary Figure 7. Single-cell transcriptomic and spatial validation reveal malignant epithelial cell-specific activation of the LDHA–H3K23la–WNK1 signaling axis and its association with an immunosuppressive tumor microenvironment in ESCC.**

**A.** Dot plot demonstrating LDHA expression levels and the proportion of LDHA-expressing cells among different cell types. **B.** UMAP visualization of major cellular populations identified by scRNA-seq. **C.** Feature plot showing LDHA expression distribution, indicating predominant enrichment in malignant squamous epithelial cells. **D.** Relative proportions of major cell populations stratified according to LDHA expression levels. **E.** Comparison of T-cell abundance between LDHA-high and LDHA-low tumors. **F.** Correlation analysis between LDHA expression and immune infiltration scores, showing enhanced adaptive immune-cell infiltration, particularly B cells and CD8⁺ T cells, in LDHA-low tumors. **G-H.** Single-cell expression analysis showing preferential expression of WNK1 (G) and AKT1 (H) in malignant epithelial cells, supporting the cellular localization of the LDHA–H3K23la–WNK1 signaling pathway. **I-J.** Multiplex immunofluorescence (mIF) validation of pathway activation and spatial immune remodeling in ESCC tissues. Representative images showing tumor regions identified by Pan-CK staining compared with adjacent Pan-CK-negative regions. Detection and quantification of LDHA and H3K23la expression in Pan-CK-positive and Pan-CK-negative regions. **K-L.** Detection and quantification of WNK1 and p-H3S10 expression in tumor and adjacent regions. Detection and quantification of proteins expression in Pan-CK-positive and Pan-CK-negative regions. **M-N.** Detection and quantification of H3K23la and p-AKT(S473) expression, demonstrating activation of the LDHA–H3K23la–WNK1–p-H3S10–AKT signaling cascade within malignant epithelial compartments. **O-P.** Spatial analysis of tumor microenvironment remodeling. Pathway-active tumor regions showed increased FAP expression and reduced CD8α⁺ T-cell infiltration, indicating an association between lactate-associated signaling activation, CAF enrichment, and immune suppression. Statistical significance was determined using a two-tailed paired Student’s t-test (J, N, L and P).

**Supplementary Figure 8. CD03017 is a potential WNK1 inhibitor.**

**A.** Molecular docking screening was performed using Autodock Vina software. Based on the analysis, the top 6 small molecules with the highest docking affinity were selected. **B.** The docking models and structural formulas of six compounds with the WNK1 protein are provided. **C.** MTT assays were performed to evaluate the effects of WNK1 inhibitors on the proliferation of ESCC cells.

**Supplementary Figure 9. CD03017 selectively suppresses ESCC cell proliferation and clonogenic growth.**

**A.** MTT assays showing that CD03017 inhibited the proliferation of ESCC cells in a dose-dependent manner, whereas no obvious inhibitory effect was observed in immortalized esophageal epithelial SHEE cells. **B-C.** Representative colony formation images and quantification showing that CD03017 significantly reduced clonogenic growth of ESCC cells in a dose-dependent manner. **D-E.** Representative soft agar colony formation images and quantification demonstrating that CD03017 dose-dependently suppressed anchorage-independent growth of KYSE30, KYSE450, KYSE150, and KYSE510 cells. **F**. Lactate production in KYSE30 and KYSE450 cells following MK-2206 treatment, showing that AKT inhibition did not significantly affect extracellular lactate levels (n=3). **G-I**. Seahorse extracellular acidification rate (ECAR) analysis showing that treatment with CD03017 (15 μM) did not significantly alter basal glycolysis or glycolytic capacity in KYSE450 cells (n=3). **J-L**. Seahorse ECAR analysis showing that pharmacological inhibition of AKT with MK-2206 (5 μM) had no significant effect on basal glycolysis or glycolytic capacity in KYSE450 cells (n=3). Data are presented as mean ± SD. Statistical significance was determined using one-way ANOVA. ns, no significant difference. Data are presented as mean ± SD from three independent experiments. Statistical significance was determined using one-way ANOVA. ns, no significant difference, **p* < 0.05; ***p* < 0.01.
