# Supplementary material for: Histone Lactylation Drives WNK1 Expression to Promote Esophageal Squamous Cell Carcinoma Progression Through Histone H3 Ser10 Phosphorylation
Source: Adv Sci (Weinh). 2026 Oct 25:e78438. Online ahead of print. doi: 10.1002/advs.78438 (PMC13696526; doi:10.1002/advs.78438)
Supplement: Supplementary file 5 [file ADVS-9999-e78438-s005.docx]

**Supplementaty Table4: Detailed clinical information of the enrolled participants**

| Personnel ID | Hospital number | Gender | Age |
| --- | --- | --- | --- |
| Patient 1 | 557521 | M | 51 |
| Patient 2 | 551350 | M | 55 |
| Patient 3 | 546908 | M | 64 |
| Patient 4 | 548647 | M | 66 |
| Patient 5 | 540642 | M | 70 |
| Patient 6 | 379904 | M | 72 |
| Patient 7 | 541680 | M | 72 |
| Patient 8 | 561941 | M | 72 |
| Patient 9 | 554300 | F | 56 |
| Patient 10 | 545805 | F | 59 |
| Patient 11 | 531690 | F | 67 |
| Patient 12 | 547999 | F | 71 |
| Patient 13 | 562693 | F | 75 |
| Patient 14 | 523904 | F | 76 |
| Patient 15 | 552436 | F | 79 |
| Normal 1 | 60862195 | M | 50 |
| Normal 2 | 60826261 | M | 54 |
| Normal 3 | 60868890 | M | 57 |
| Normal 4 | 60810896 | M | 61 |
| Normal 5 | 60856853 | M | 66 |
| Normal 6 | 6005155 | M | 71 |
| Normal 7 | 9008969 | M | 71 |
| Normal 8 | 6005155 | M | 71 |
| Normal 9 | 60810150 | F | 53 |
| Normal 10 | 60868898 | F | 58 |
| Normal 11 | 60858858 | F | 67 |
| Normal 12 | 60860356 | F | 72 |
| Normal 13 | 60862217 | F | 73 |
| Normal 14 | 9000172 | F | 75 |
| Normal 15 | 958680 | F | 75 |
