# Supplementary material for: Histone Lactylation Drives WNK1 Expression to Promote Esophageal Squamous Cell Carcinoma Progression Through Histone H3 Ser10 Phosphorylation
Source: Adv Sci (Weinh). 2026 Oct 25:e78438. Online ahead of print. doi: 10.1002/advs.78438 (PMC13696526; doi:10.1002/advs.78438)
Supplement: Supplementary file 6 [file ADVS-9999-e78438-s006.docx]

**Supplementaty Table2: Kinase profile**

| Kinase name | Catalogue Number | WI @ 1.0 µm(Activity %） |
| --- | --- | --- |
| Aurora-A(h) | 14-511KP | 112 |
| B-Raf(h) | 14-530KP | 104 |
| GSK3β(h) | 14-306KP | 103 |
| JAK1(h) | 14-918KP | 104 |
| MAPK3(h) | 14-439KP | 104 |
| MEK1(h) | 14-429KP | 97 |
| mTOR(h) | 14-769KP | 94 |
| NEK2(h) | 14-545KP | 108 |
| Src(1-530)(h) | 14-746KP | 103 |
| PKBα(h) | 14-276KP | 111 |
| PKBβ(h) | 14-339KP | 105 |
| PKBγ(h) | 14-502KP | 109 |
| Rsk1(h) | 14-509KP | 99 |
| STK39(h) | 16-049KP | 99 |
| OSR1(h) | 16-048KP | 111 |
| PI3 Kinase (p110α(E542K)/p85α)(h) | 14-782KP | 102 |
| WNK1(h) | 16-007KP | 79 |
| WNK2(h) | 14-678KP | 103 |
| WNK3(h) | 14-658KP | 97 |
| WNK4(h) | 16-047KP | 104 |
