# Supplementary material for: Histone Lactylation Drives WNK1 Expression to Promote Esophageal Squamous Cell Carcinoma Progression Through Histone H3 Ser10 Phosphorylation
Source: Adv Sci (Weinh). 2026 Oct 25:e78438. Online ahead of print. doi: 10.1002/advs.78438 (PMC13696526; doi:10.1002/advs.78438)
Supplement: Supplementary file 7 [file ADVS-9999-e78438-s007.docx]

**Supplementary Table 1-1. Cox regression analysis of H3K23la expression and overall survival**

| Variable | Univariate Cox regression analysis | | Multivariate Cox regression analysis | |
| --- | --- | --- | --- | --- |
|  | HR（95%CI） | *P*-value | HR（95%CI） | *P*-value |
| Age | 1.018（0.992–1.045） | 0.179 | 1.015（0.987–1.044） | 0.299 |
| Male vs Female | 1.900（0.969–3.725） | 0.062 | 1.790（0.884–3.624） | 0.106 |
| Histological grade | 0.620（0.395–0.973） | 0.038 | 0.680（0.418–1.106） | 0.120 |
| T stage | 2.680（1.455–4.937） | 0.002 | - | - |
| N stage | 1.710（1.255–2.329） | <0.001 | - | - |
| AJCC stage | 3.090（1.784–5.352） | <0.001 | 2.650（1.450–4.844） | 0.002 |
| H3K23la expression (high vs low) | 1.688（1.038–2.746） | 0.035 | 1.880（1.118–3.162） | 0.017 |

**Supplementary Table 1-2. Cox regression analysis of WNK1 expression and overall survival**

| Variable | Univariate Cox regression analysis | | Multivariate Cox regression analysis | |
| --- | --- | --- | --- | --- |
|  | HR（95%CI） | *P*-value | HR（95%CI） | *P*-value |
| Age | 1.000（0.973–1.027） | 0.990 | 0.995（0.966–1.025） | 0.750 |
| Male vs Female | 1.520（0.807–2.862） | 0.196 | 1.320（0.681–2.558） | 0.411 |
| Histological grade | 0.780（0.493–1.235） | 0.289 | 0.750（0.459–1.226） | 0.252 |
| T stage | 2.780（1.490–5.186） | 0.001 | - | - |
| N stage | 1.790（1.314–2.440） | <0.001 | - | - |
| AJCC stage | 3.220（1.884–5.502） | <0.001 | 3.606（1.994-6.519） | <0.001 |
| WNK1 expression (high vs low) | 2.162（1.275–3.666） | 0.004 | 2.017（1.147–3.546） | 0.015 |

**Supplementary Table 1-3. Cox regression analysis of P-Histon H3 S10 expression and overall survival**

| Variable | Univariate Cox regression analysis | | Multivariate Cox regression analysis | |
| --- | --- | --- | --- | --- |
|  | HR（95%CI） | *P*-value | HR（95%CI） | *P*-value |
| Age | 1.021（0.998–1.045） | 0.076 | 1.017（0.992–1.043） | 0.185 |
| Male vs Female | 1.590（0.870–2.906） | 0.132 | 1.510（0.800–2.850） | 0.203 |
| Histological grade | 0.680（0.449–1.031） | 0.069 | 0.730（0.464–1.148） | 0.173 |
| T stage | 2.750（1.625–4.654） | <0.001 | - | - |
| N stage | 1.820（1.380–2.400） | <0.001 | - | - |
| AJCC stage | 3.980（2.388–6.633） | <0.001 | 3.320（1.880–5.862） | <0.001 |
| P- Histone H3 S10 expression (high vs low) | 2.188（1.388–3.452） | <0.001 | 1.733（1.039–2.890） | 0.035 |
